# Influence of surface characteristics on the in vitro stability and cell uptake of nanoliposomes for brain delivery

**DOI:** 10.3762/bjnano.17.9

**Published:** 2026-01-13

**Authors:** Dushko Shalabalija, Ljubica Mihailova, Nikola Geskovski, Andreas Zimmer, Otmar Geiss, Sabrina Gioria, Diletta Scaccabarozzi, Marija Glavas Dodov

**Affiliations:** 1 Ss. Cyril and Methodius University in Skopje, Faculty of Pharmacy, Institute for Pharmaceutical Technology, Mother Theresa 47, 1000 Skopje, N. Macedonia; 2 University of Graz, Institute of Pharmaceutical Sciences, Department of Pharmaceutical Technology and Biopharmacy, Universitatplatz 1/EG, A-8010, Graz, Austria; 3 European Commission, Joint Research Centre (JRC), Ispra, Italy

**Keywords:** blood–brain barrier, cell co-culture, cell uptake, internalization, nanoliposomes, stability, surface characteristics

## Abstract

In contemporary research, there is a clear emphasis on the physicochemical characteristics and effectiveness of nanoliposomal (NLs) formulations. However, there has been minimal focus on elucidating nano–bio interactions and understanding the behavior of these formulations at organ and cellular levels. Specifically, it is widely recognized that when exposed to biological fluids, nanodelivery systems, including NLs, rapidly interact with various biomolecules which have a significant impact on the functionality and fate of the nanosystems but also influence cellular biological functions. Hence, the primary objective of this study was to elucidate the evolution of physicochemical characteristics and surface properties of NLs in biorelevant media. Additionally, in order to point out the influence of specific characteristics on the brain targeting potential of these formulations, we investigated interactions between NLs and blood–brain barrier (BBB, hCMEC/D3) and neuroblastoma cells (SH-SY5Y) under different conditions. The results obtained from comparative in vitro cell uptake studies on both cell culture lines after treatment with three different concentrations of fluorescently labelled NLs (5, 10, and 100 μg/mL) over a period of 1, 2, and 4 h showed a time- and concentration-dependent internalization pattern, with high impact of the surface characteristics of the different formulations. In addition, transport studies on hCMEC/D3/SH-SY5Y co-cultures confirmed the successful transport of NLs across the BBB cells and their subsequent uptake by neurons (ranging from 25.17% to 27.54%). Fluorescence and confocal microscopy micrographs revealed that, once internalized, NLs were concentrated in the perinuclear cell regions.

## Introduction

Advancements in nanotechnology and the use of nanoliposomes (NLs) as carriers for targeted delivery and controlled release of active components (AC) show promise in addressing multiple pathologies associated with neurodegenerative diseases like Alzheimer's disease (AD) [1]. It is well known that incorporating AC into NLs improves their biological distribution, reduces macrophage uptake, and lowers free AC concentrations, thus decreasing systemic toxicity. Additionally, modifying the composition and surface properties of NLs can enhance their passage through the blood–brain barrier (BBB) and target specific brain regions [2–3]. However, despite these advantages, only few NLs formulations for brain diseases have completed clinical trials and are commercially available [4]. Among other limitations, one of the most underestimated steps especially in early formulation stages may stem from the incomplete understanding of factors affecting the NLs optimal delivery to the central nervous system (CNS). Therefore, understanding the mechanisms of NLs transport across the BBB and the impact of NLs properties as well as the disease pathophysiology is crucial for improving therapeutic outcomes [3,5].

Namely, current research on NLs predominantly focuses on their physicochemical characteristics and efficiency, but minimal attention has been given to understanding the nano–bio interactions at organ and cellular levels. When exposed to biological fluids, nanodelivery systems like NLs interact with biomolecules, thus forming a protein corona (PC), altering functional proteins, and engaging in redox reactions with reactive species [6]. These interactions can affect the functionality, biodistribution, targeting, and cell internalization of NLs. Serum components can disrupt the lipid structure of NLs, leading to AC leakage, while plasma protein adsorption may cause particle aggregation [7]. In this sense, the presence of proteins in the tissue environment can alter cellular uptake of both cationic and anionic carriers [8]. In short, our understanding of how nanodelivery systems behave in biological environments is limited, and further research is needed to explore the fundamental mechanisms of nano–bio interactions and develop strategies for their manipulation.

In order to obtain valuable insights into the behavior of nanodelivery systems, as well as the nano–bio interactions in complex organs like the brain, in vitro cell culture models, such as hCMEC/D3 and SH-SY5Y, are often used [9]. Therefore, the hCMEC/D3 model is useful for examining nanosystems, drug uptake, and transport across the BBB, with advantages like easy growth and mimicking basic BBB properties. However, optimizing tight junctions to mirror human BBB characteristics remains a challenge [10–11]. On the other hand, the SH-SY5Y neuroblastoma cell line, which can further differentiate into neuronal-like morphologies, is often used to evaluate nanodelivery systems uptake kinetics and drug efficacy especially in the context of brain-related disorders such as Alzheimer's and Parkinson's, thus providing useful starting point for testing potential therapies and understanding disease mechanisms [12–13]. Hence, proper cell line selection and optimized experimental conditions help predicting in vivo stability, toxicity, and therapeutic potential of nanodelivery systems, thus improving the translation of study results to real biological systems. In this direction, some of the prerequisites for establishing a relevant in vitro model for cell uptake studies include precise control over factors like endothelial monolayer integrity and permeability of hCMEC/D3 and SH-SY5Y in order to accurately simulate NLs uptake across the BBB and to obtain accurate representation of neuronal uptake mechanisms [14]. Other factors playing a key role in maximization of the uptake efficiency and prevention of saturation or incomplete uptake, are suitable adjustments in the incubation time, temperature, and NL concentration, as well as pH and ion concentrations. Optimization of these factors is essential for accurate identification of transport mechanisms, like diffusion or receptor-mediated endocytosis, also reflecting the true dynamics of NLs and drug absorption or trafficking within brain cells. Finally, optimized experimental conditions help reduce variability between cell uptake experiments and ensure that results are consistent across different cell passages, laboratories, or research conditions which is particularly important when conducting studies that will later be informative for clinical or therapeutic applications [15–16].

It is important to emphasize that the literature data on the physical stability of NLs during their passage through different physiological barriers and compartments is limited. Additionally, the information regarding the influence of different experimental conditions on the NL cell uptake outcome in brain cell culture lines is also scarce. In our previous work, we compared nanostructured lipid carriers (NLCs) and liposomes modified with different stealth polymers (poloxamer or poly(ethylene glycol) with a fixed concentration different than that used in the present study), examining their cellular uptake at a single time point. The results highlighted that both the type of nanocarrier and the nature of the surface polymer critically influence nanoparticle–cell interactions, including the internalization pathway and intracellular trafficking [17]. In contrast, this study focuses on the evolution of physicochemical and surface properties of NLs in biorelevant media depending on the different amount of PEG onto NLs surface, thus correlating these findings with the cell internalization efficacy. The study also aimed to explore how these characteristics influence brain accumulation by examining interactions with different cell cultures under varying experimental conditions. In this context, time- and concentration-dependent uptake experiments were conducted on hCMEC/D3 and SH-SY5Y cell lines to predict how formulation properties and the extracellular microenvironment affect the in vivo performance of NLs, and access transport mechanisms as well as internalization in brain tissues. Additionally, transport experiments on a co-culture system (hCMEC/D3/SH-SY5Y) were performed to confirm efficient NLs transit across the BBB and successful uptake into neurons.

In summary, optimizing experimental conditions is essential for producing reliable, biologically relevant data in cell uptake studies, as well as to confirm that the results reflect true biological processes, reducing artifacts and avoiding false positives.

## Materials and Methods

### Materials

Soybean lecithin (SL) was purchased from Vitalia (Skopje, N. Macedonia) and LIPOID PE 18:0/18:0-PEG 2000 (PEG) from Lipoid (Ludwigshafen am Rhein, Germany). Cholesterol (CH), Dulbecco’s Phosphate Buffered Saline (DPBS), collagen type I from rat tail, fluorescein isothiocyanate isomer I (FITC), and Nile red dye were obtained from Sigma-Aldrich (St. Louis, USA). Immortalized human cerebral microvascular endothelial (hCMEC/D3) cell line (CELLutions, Biosistems/Cedarlane^®^, Canada) were maintained in Endothelial Basal Medium-2 (EBM-2), supplemented with fetal bovine serum (FBS), chemically defined lipid concentrate, HEPES 1M, and penicillin−streptomycin (Life Technologies, California, USA), human basic fibroblast growth factor (bFGF), ascorbic acid, and hydrocortisone (Sigma-Aldrich, St. Louis, USA) and detached with trypsin-EDTA (GIBCO, Thermo Fisher Scientific, California, USA). The human neuroblastoma cell line (SH-SY5Y) was purchased from LCG Standards (Wesel, Germany) and maintained in Dulbecco's Modified Eagle Medium (DMEM, Thermo Fisher Scientific, California, USA). EndoGRO-MV SCME004 complete media kit was provided by Merck (Merck Group, Germany). CellTiter 96 AQueous Non-Radioactive Cell Proliferation Assay (MTS) and CytoTox-ONE^TM^ Homogeneous Membrane Integrity Assay (CytoTox) were obtained from Promega (Wisconsin, USA). Alexa Fluor^TM^ Phalloidin 488, Hoechst fluorescent stain and Dil stain (1,1'-dioctadecyl-3,3,3',3'-tetramethylindocarbocyanine perchlorate ('DiI'; DiIC18(3))) were purchased from Thermo Fisher Scientific (California, USA).

All chemicals and reagents used were of the highest purity grade commercially available.

### Preparation of the nanoliposomes

Nanoliposome samples (SL:CH:PEG = 8.71:1:0; 8.71:1:1.67, and 8.71:1:0.67 molar ratios for NLb0, NLb1, and NLb2, respectively) were prepared by modified lipid film hydration technique as described in our previous work [18]. The chosen formulations used for these studies were previously optimized and underwent complete characterization, where it was shown that the used amounts of PEG drastically affect the physicochemical, biopharmaceutical, and surface characteristics of the vesicles.

For this process, the required amounts of SL, CHOL, and commercially available mPEG2000 conjugated to DSPE (PEG) were dissolved in a methanol/chloroform mixture (1:4, v/v). The organic solvents were then evaporated under vacuum using a rotary evaporator (25 °C, 50 rpm, 50 mbar; Büchi 215, Switzerland). The resulting thin lipid film was hydrated with phosphate buffer (PB) at pH 7.4. The hydration process involved four consecutive cycles, each consisting of three 5 min steps: ultrasonication (50/60 Hz; ULTRASONS-H, J.P. Selecta), vortexing (Tehtnika, EV-102, Slovenia), and manual mixing at room temperature. The prepared liposomes underwent high shear homogenization (24,000 rpm for 5 min; Ultra-Turrax T25, IkaWerke, Germany) and were then incubated at 4–8 °C for 24 h. Finally, the liposomal dispersion was homogenized again at 6,000 rpm for 3 min and stored at 4–8 °C.

All fluorescent dyes, Nile red (1.6%, w/w) and Dil stain (2.5%, w/w), used in cell culture experiments were added in the organic phase during the preparation process, according to Mihailova et al. [17]. In order to remove the unincorporated fluorophore dye, NL dispersions were centrifuged (Rotofix 32 - Hettich Zentrifugen, Germany) at 4,500 rpm, 25 °C, 15 min, four cycles in Vivaspin 20 ultrafiltration cuvettes, 100,000 MWCO units (Sartorius, Germany) with subsequent supernatant removal. Furthermore, the removal of unencapsulated dye was qualitatively confirmed by spectrophotometry (e.g., by the absence of fluorescence in the supernatant obtained in the last washing cycle), ensuring that the fluorescence signal in subsequent experiments originates exclusively from liposome-associated dye.

### Particle size, particle size distribution and *z*-potential of the nanoliposomes

Тhe *z*-average diameter, the polydispersity index (PDI), and the *z*-potential (ZP) were determined using a Zetasizer Nano Series, (Nano-ZS, Malvern Instruments Ltd., UK), after diluting the optimal NL samples in 10 mM PB pH 7.4 (1:20, v/v). Measurements were made under the following conditions: 25 °C, thermostating time of 120 s, viscosity of the medium 0.8894 cP, dielectric constant of 78.5, and an angle of 173°. At least three separate preparations (batches) from each sample were measured in triplicate. The results of each analysis were the average of 12 consecutive measurements.

### Stability studies of nanoliposomes in cell culture medium

The samples (NLb0, NLb1, and NLb2) were first diluted to a final concentration of 1 mg/mL (total volume of 2 mL) in cell culture medium (Endo-GRO-MV, Cat SCME004, Sigma-Aldrich), and then incubated at 37 °C for 1 and 4 h in an Eppendorf thermomixer under constant stirring (300 rpm). The incubation took place in serum free and 5% serum-supplemented cell culture medium (prepared according to the instructions from the manufacturer). At the end of the incubation time, the samples were vortexed (5 s) and a volume of 1 mL was transferred to a vial for asymmetric flow field-flow fractionation analysis (AF4 analysis). Experiments were carried out by in-line coupling the AF4 system with UV–vis, multi-angle light scattering (MALS), and dynamic light scattering (DLS) detectors.

The AF4 system used was composed of an Eclipse Dualtec separation system (Wyatt Technology Europe GmbH, Dernbach, Germany) and an Agilent 1260 Infinity high-performance liquid chromatograph (Agilent Technologies, Santa Clara, USA) equipped with a degasser (G1322A), an isocratic pump (G1310B), an autosampler (G1329B), and a multiple-wavelength detector (MWD, G1365C) set at 230 nm. In the Eclipse SC separation channel, regenerated cellulose membranes (10 kDa) and a spacer height of 350 μm were used. Phosphate buffer was used as the eluent. The detector flow rate was set to 0.5 mL/min and the injection volume was 50 µL. The separation settings were: a) elution 0–3 min; b) focus: 3–5 min (focus flow: 1.0 mL/min); c) focus + injection: 5–10 min (focus flow: 1.0 mL/min); d) elution: 10–50 min (cross-flow: 1.0–0.1 mL/min); e) elution: 50–55 min (cross-flow: 0.1 mL/min); f) elution: 55–60 min (cross-flow: 0.1–0.0 mL/min); g) elution: 60–70 min (cross flow: 0.0 mL/min). The outlet of the MWD detector was connected to a DAWN 8+ HELEOS II MALS operating with a 658 nm laser (Wyatt Technology Europe). A DLS (Malvern Zetasizer Nano-S, UK) with an installed quartz flow cell (ZEN0023) was also used in this study in flow mode. Refractive index (RI) and absorption parameters were set to 1.38 and 0.010, respectively. Water was set as the dispersant, the temperature was set to 25 °C, while the attenuation was set to 11. The 'general purpose (normal distribution)' was chosen as the analysis model.

### High-resolution automated electrophoresis of the adsorbed proteins onto nanoliposomal surface

Protein electrophoresis was performed by a 2100 Bioanalyzer using High Sensitivity Protein 250 kit assays in reducing condition according to the instruction from the manufacturer. Briefly, samples were prepared as for the AF4 analysis. At the end of the incubation time, samples were vortexed for 5 s and centrifuged in an Eppendorf centrifuge (Eppendorf 5810R (Hamburg, Germany)) at 13.000 rpm at room temperature for 30 min. All pellets were then washed three times with 1 mL of PBS (Gibco, Cat AM9624), and after each wash the supernatant was collected. Next, samples with pellets were diluted 200-fold in milli-Q water and denatured at 95 °C for 5 min in reducing condition by adding 3.5 µL of 1 M dithiothreitol (DTT) buffer solution to 100 µL of each sample buffer. After cooling, samples were loaded on the microfluidic chip for electrophoresis, in accordance with the instructions from the manufacturer. All reagents and instruments were from Agilent Technology, California, USA.

#### hCMEC/D3 and SH-SY5Y cell culture lines

Both cell culture lines were seeded and cultivated according to the guidelines from the supplier, explained in details in Mihailova et al. [17].

In vitro uptake and internalization experiments were conducted using hCMEC/D3 cell cultures (passages 21–25). T-75 cell culture flasks (Greiner Bio-One GmbH, Germany) were initially coated with 0.05 mg/mL of rat tail collagen type I in DPBS and left to stand for at least 1 h at 37 °C. The cells were cultured in supplemented EBM-2 at 37 °C with 5% CO_2_. Medium replacement occurred every 2–3 days until the cells reached confluence. Upon reaching confluence, the medium was aspirated, and the cells were detached from the flask walls by incubating them at 37 °C for 8 min in 0.1 mg/mL of trypsin–EDTA solution. The cell suspension was then centrifuged at 1500 rpm for 3 min, and the supernatant was discarded. The cells were resuspended in 5 mL of cell medium and were prepared for further experimentation.

The human neuroblastoma cell line (passages 14–17) was cultured in DMEM at 37 °C in a 5% CO_2_ atmosphere. Medium replacement occurred every 2–3 days until cells reached confluence. During the splitting process, the medium was aspirated, and the cells were detached from the flask walls after incubating at 37 °C for 3 min in 0.1 mg/mL trypsin–EDTA solution. The cell suspension was then centrifuged at 800 rpm for 5 min, and the supernatant was discarded. The cells were subsequently resuspended in 5 mL of cell medium, making them ready for further experiments.

### Cell uptake assessment of nanoliposomes

In order to determine the influence of the PEG amount and exposure times on the uptake of the NLs, hCMEC/D3 or SH-SY5Y cells were seeded onto 96-well plates at a density of 10^4^ cells/well in their respective cell culture medium (200 μL/well) and incubated for 48 h at 37 °C in the presence of 5% CO_2_. As previously described, wells for hCMEC/D3 cells were pre-coated with collagen type 1 (0.05 mg/mL) for 1 h. After reaching confluence, the cell culture medium was replaced and the cells were treated with different concentrations of the fluorescent dye labeled formulations NLb0, NLb1, and NLb2 labelled with Nile red (Sigma-Aldrich, USA, previously diluted with PBS and dispersed in the appropriate cell culture medium at final NLs concentrations of 5, 10 or 100 μg/mL). Incubation was performed for 1, 2, and 4 h (37 °C, 5% CO_2_), followed by a washing step with PBS and lysis with 2% Triton X-100 (2 h, 37 °C, 5% CO_2_). The resulting fluorescence was measured on a plate reader at an excitation wavelength of 535 nm and an emission wavelength of 635 nm (BMG Labtech, Ortenberg, Germany). Untreated cells were used as blank. The quantitative amount of internalized NLs was calculated according to pre-obtained regression analysis equations obtained from the fluorescence gotten over a range of concentrations for each of the formulations (nonincubated with cells). The experiments were performed three times on at least six replicates from each sample.

### Cell uptake assessment of nanoliposomes in the presence of transport pathways inhibitors

In order to determine the exact mechanism of cellular internalization, uptake experiments were also performed in the presence of inhibitors for specific transport pathways. Cells were seeded and cultured as described above.

The quantitative amount of the internalized NLs was investigated on both cell lines. One set of cells was treated with 15 μM of chlorpromazine and another set with 25 μM of indomethacin for 40 min, followed by incubation with Nile red labelled NLs (at final NLs concentrations of 5, 10, or 100 μg/mL) for 2 h (37 °C, 5% CO_2_). The third set of cells was left at 4 °C for 40 min, before incubation of NLs in cold condition for 2 h. The resulting fluorescence was measured as previously described (same conditions as for the 37 °C experiments), and the quantitative amount of internalized NLs was calculated and expressed as the % of the uptake of the corresponding NL concentrations at 37 °C (taken as 100%). This was done in order to facilitate interpretation and eliminate inter-experimental variability (e.g., in cell number, fluorescence intensity calibration, or liposome batch differences) as well as to directly compare the relative contribution of each endocytic pathway to the overall internalization process. The experiments were performed three times on six replicates from each sample.

### Cell uptake experiments on co-cultured hCMEC/D3 and SH-SY5Y cell lines

The hCMEC/D3 cells were seeded onto Transwell inserts (5 × 10^4^ cells/insert), previously coated with 0.05 mg/mL type I collagen in DPBS (1 h, 37 °C) and incubated with EBM-2 (0.5 mL of cell medium in the apical part of the insert and 1 mL in the basal part of the well). The medium was changed every 2–3 days until a transendothelial resistance (TEER) value >230 Ω was reached, indicative of confluent monolayer formation and tight junctions between endothelial cells [11]. In parallel, SH-SY5Y cells were seeded onto 12-well plates (3 × 10^4^ cells/well) and cultured in 1 mL DMEM for the same period of time, until reaching confluence. Furthermore, the inserts with hCMEC/D3 cells were placed in the plates with formed monolayers of SH-SY5Y. Nile red labelled NLs previously diluted in PBS and dispersed in EBM-2 (final NL concentration of 10 μg/mL) were added to the apical part of the inserts, and 1 mL of DMEM was added to the basal part of the plates. After 2 h of incubation (TEER >210 Ω), the inserts were removed and the SH-SY5Y wells were washed twice with PBS and lysed with 2% Triton X-100 (2 h, 37 °C, 5% CO_2_). Any potential retention of NLs within the Transwell insert was minimized by gentle mixing. The resulting fluorescence was measured on a plate reader at a 535 nm excitation and 635 nm emission wavelengths (BMG Labtech, Ortenberg, Germany). The wells with SH-SY5Y cells incubated in the corresponding cell medium (not treated with NLs) were used as blanks. Regarding uptake quantification, the % of uptake was determined fluorometrically, based on the fluorescence intensity of internalized, Nile-red-labeled NLs normalized to the total amount applied to the culture. Additionally, prior to all cell uptake experiments, a standard curve of Nile-red-labelled NLs was prepared (incubated under the same conditions, without cells). The experiments were performed three times on at least six replicates from each sample.

### Internalization studies

#### Internalization studies of nanoliposomes in live hCMEC/D3 and SH-SY5Y cell lines by fluorescence microscopy

In order to obtain a deeper insight of the internalization and co-localization of the prepared NLs samples in living cells, hCMEC/D3 and SH-SY5Y cells were first seeded onto 35 mm glass dishes at a density of 2 × 10^5^ cells per well (μ-Dish 35 mm, WillCo Glass Bottom Dishes, Netherlands) and a subsequent incubation for 48 h (37 °C and 5% CO_2_) was conducted. Afterwards, the medium was removed, and the cells were treated with Nile-red pre-labeled NLs (NLb0, NLb1, and NLb2) dispersed in the respective cell culture medium at a final NL concentration of 10 μg/mL. After 1, 2, and 4 h of incubation (37 °C, 5% CO_2_), the cells were washed twice with PBS, followed by subsequent fluorescence microscopy analysis at 37 °C (Zeiss Axio Observer Z1 inverted microscope, Zeiss, Jena, Germany), equipped with an epifluorescence illuminator and a plate heating chamber. The resulting images were processed using the Carl Zeiss software (ZEN 2.6).

In this direction, another set of experiments was conducted, where SH-SY5Y cells before the incubation with pre-labeled NLs with Dil (10 μg/mL, 4 h, 37 °C, 5% CO_2_), were incubated with pHrodo Green dextran conjugate characterized by green fluorescence in an acidic environment (for visualization of endocytic pathways). Further, the cells were stained and visualized as described above.

#### Internalization studies of nanoliposomes in hCMEC/D3 cells by confocal microscopy

Confocal laser scanning microscopy (Carl Zeiss, Axiovert 200M Inverted Microscope) was performed in order to confirm the internalization of NLs and their co-localization in cellular structures of the BBB. For this purpose, cells were seeded onto 35 mm glass dishes (μ-Dish 35 mm, WillCo Glass Bottom Dishes, Netherlands) at a density of 2 × 10^5^ cells per well and incubated for 24 h (37 °C and 5% CO_2_). After incubation (4 h, 37 °C and 5% CO_2_) with the previously labeled NLs with the Dil fluorescent dye, the cells were washed twice with PBS and fixed with 3.7% paraformaldehyde for 20 min at room temperature. After washing the cells with PBS, the cytoskeleton was stained with Alexa Fluor^TM^ Phalloidin 488 green (6.6 μM) in 1% bovine serum albumin (10 min, 37 °C), followed by nuclear staining with Hoechst (hCMEC/D3), for 5 min at room temperature. Before microscopic visualization, the Vectashield mounting medium was added to maintain and preserve the fluorescent dye. Images were processed using the Carl Zeiss software (ZEN 2.6).

### Statistical analysis

Statistical analysis of the obtained results for quantitative uptake of NLs was carried out by implementing the method of least squares (PLS) using the validated statistical software Simca 14.1 (Sartorius Stedim Biotech, Germany). In order to highlight the dominant independent variables that have a significant effect in the model, the VIP score was used.

## Results and Discussion

### Particle size, particle size distribution and *z*-potential of the nanoliposomes

Several studies have reported that despite the composition and the other surface properties (i.e. surface charge), the size range of long time circulating NPs may strongly affect their stability, in vivo circulation time, as well as BBB retention, and the possibility of entering into specific interactions with the BBB structures, thus their transport and uptake by brain cells [19]. In this direction, one of the initial steps was the determination of the *z*-average diameter and *z*-potential of NLs in order to further correlate them to the outcome of cell uptake experiments.

The *z*-average (hydrodynamic) diameter of the prepared NLs formulations with different amounts of PEG coated on the surface ranged from 115–130 nm with PDI < 0.3 (Table 1), which indicates a narrow unimodal size distribution of the NLs in all formulations. According to the literature data, liposomal nanovesicles from 100 to 140 nm exhibit longer half-life in blood circulation and avoid of PC formation when compared those of nanovesicles with diameter >200 nm, and also, have better encapsulation efficiency than liposomes <100 nm [20]. On the other hand, in the study of Nowak et al. [21] it was confirmed that spherical particles around 120 nm associate with the endothelium approximately 30‐fold more than 200 nm particles, which is of extreme importance for their successful transport across the BBB and consequently, efficient treatment of CNS diseases.

**Table 1:** Physical characterization of prepared NLs (*n* = 6).

formulation	*z*-average [*d*_h_, nm]	PDI	zeta potential [mV]

NLb0	127.25 ± 0.12	0.277 ± 0.00	−51.96 ± 2.22
NLb1	114.90 ± 0.92	0.238 ± 0.00	−15.77 ± 0.51
NLb2	131.03 ± 0.30	0.289 ± 0.04	−37.64 ± 1.42

From Table 1, a decreasing trend of the negative *z*-potential with the increase of the PEG amount on the surface of NLs can also be observed. As previously reported in our earlier study, this reverse trend is probably a result of the reduced NL electrophoretic mobility due to the hydrodynamic resistance given by the presence of PEG. This also contributes for masking the predominant negative charge of the structural phospholipids present in the NLs [18].

### Stability studies of nanoliposomes in cell culture medium

In order to provide a more detailed examination of the influence of serum components present in the cell culture medium on the average NLs size, AF4–MALS/DLS analysis was performed. For this purpose, the particle size of the native formulations (NLb0, NLb1, and NLb2) was first determined, as well as after their incubation with serum-free and serum-supplemented cell culture medium over 1 and 4 h. Prior to performing these fractionation analyses, the z-average mean diameter of the NLs was also examined by dynamic light scattering in batch mode, and ranged from 96.10 ± 0.81 to 140.20 ± 0.95 nm (PDI < 0.256).

The data in the literature suggests that DLS is widely employed for sizing liposomes and other colloidal materials. It operates by detecting laser light scattered due to the Brownian motion of particles or macromolecules in suspension, with the scattering frequency dependent on particle size, offering rapid and straightforward analyses. However, larger particles can skew results and complicate measurements in heterogeneous samples, typically requiring a five-fold difference in average size to resolve distinct populations. In contrast, AF4 is more time intensive but excels in fractionating samples under optimal flow and separation conditions. This method is distinguished by gentle separation conditions and a wide operational range. Unlike conventional chromatography, AF4 does not utilize a stationary phase. The separation mechanism involves a longitudinal parabolic flow profile within the channel. This profile induces smaller particles to elute more swiftly compared to larger particles, particularly in proximity to the semipermeable membrane [22]. In particular, AF4 is a precise method for separating liposomes based on their hydrodynamic size, with particle sizes determined directly from their elution times. AF4–MALS has been extensively utilized for sizing various categories of nanoparticles such as metal oxides, polymeric and silica nanoparticles. Additionally, it has been employed for the separation of diverse macromolecules and structures including proteins, viruses, and cells. These applications have facilitated the analysis of liposomes, enabling the separation of populations obtained from the same method synthesis and determination of their size. Optimizing separation variables in AF4–MALS involves several parameters such as cross-flow conditions, focusing rate and duration, sample loading, and carrier conditions. The composition of the carrier buffer, as well as its ionic strength and pH are crucial considerations for stabilizing the structures, preventing agglomeration or sedimentation, and avoiding interference with analytes and the membrane [23].

**Figure 1:**
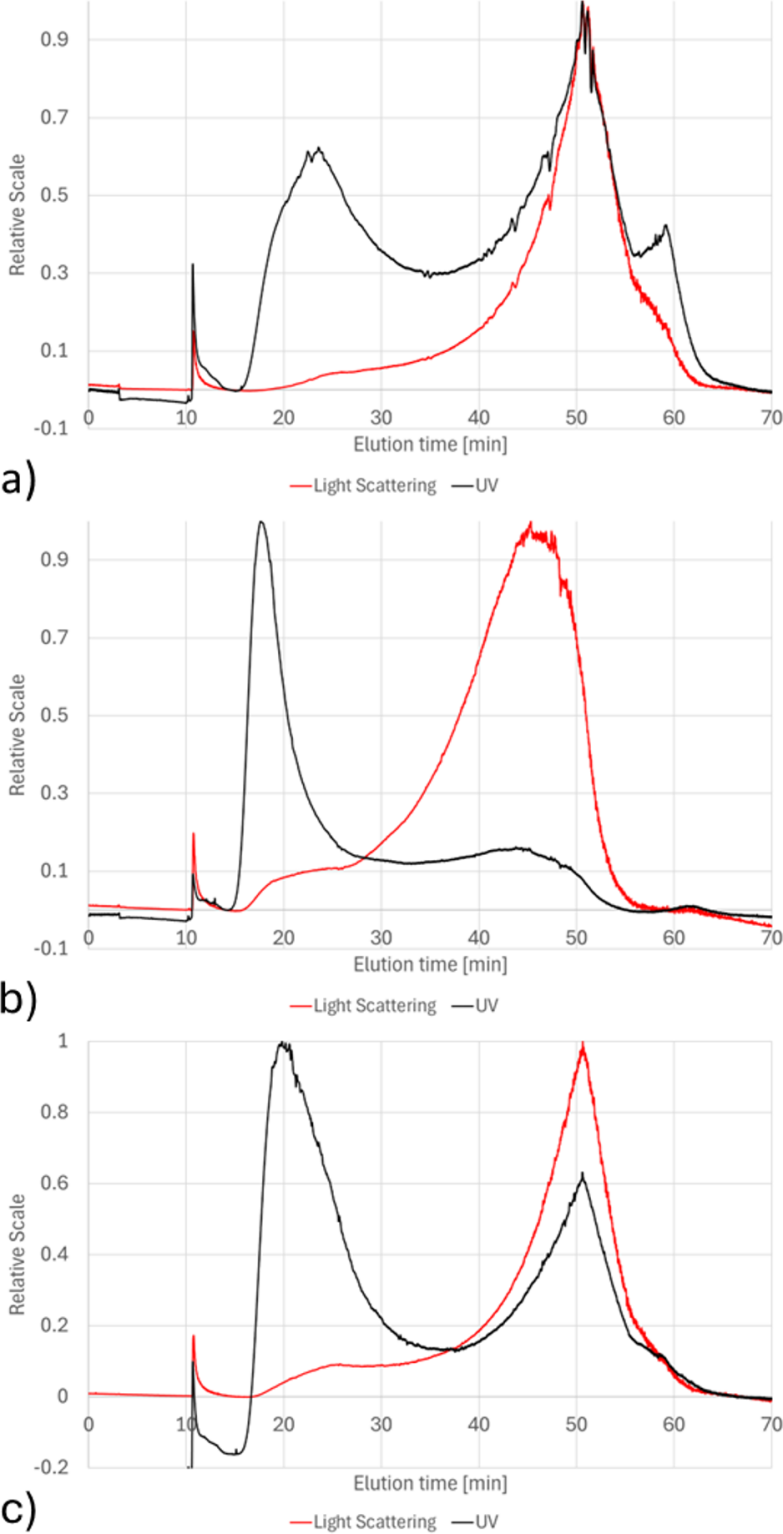
Fractograms from the qualitative evaluation using AF4–UV–MALS–DLS of the native formulations a) NLb0, b) NLb1, and c) NLb2.

Figure 1 represents the fractograms obtained from the AF4 analysis of the native formulations. The black line originates from the UV signal (230 nm) and the red line from the light scattering signal at 90°. Both signals were normalized to the highest signal. The UV-absorbance peaks (black signal), observed at a retention time of about 20 min, are most likely related to the absorption of the PEG present on the surface of NLs. In addition, it can be seen that a more intense absorption peak is obtained at the same retention time for NLb1, the formulation with the highest amount of PEG on the surface, in comparison to NLb2. Moreover, it can be observed that the shape of the light-scattering (red line) and UV signals (RT 40–60 min) of those materials treated with little or no PEG (NLb0 and NLb2) looks differently compared to that of NLb1 (pyramid-like vs near-Gaussian-like). As already mentioned in several occasions, the presence of PEG on the surface of NLs can improve the physical stability of the liposomal dispersions through steric repulsion [24]. Therefore, formulations with a low amount of PEG or no PEG tend to agglomerate or lose their native structure, thus leading to fragmentation. Hence, the unusual peak shape at 20 min and the weak signal at around 60 min in NLb0 may be a result of the absorption of various fragments of the nanoliposomes and/or some of the components present in the soybean lecithin, as well as artefacts of the initial formation of peroxides in the unsaturated fatty acid residues of the phospholipid molecules which show maximum absorbance at around 230 nm [25].

In Supporting Information File 1, Figure S1, the light scattering signal at 90° and the related geometrical radii are overlaid for native formulations NLb0, NLb1, and NLb2. The formulation NLb1 (red lines) showed to contain slightly larger particles in the final part of the eluted peak compared to the other two formulations, probably attributable to some small, insignificant fraction of agglomerated NLs formed during the measurements. The same was confirmed by DLS in-line measurements (Supporting Information File 1, Figure S2).

**Figure 2:**
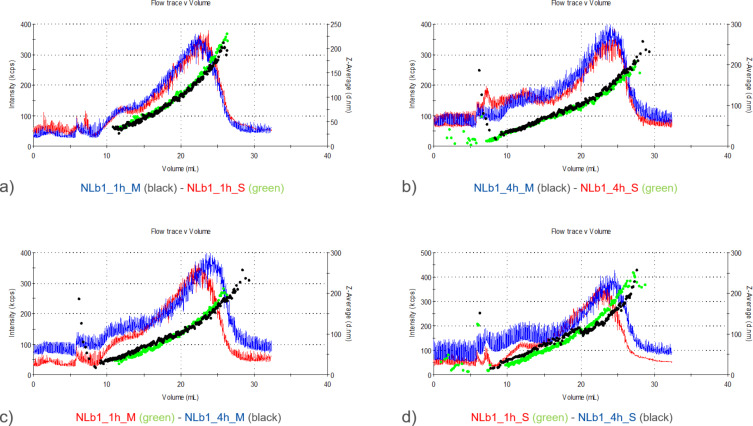
Comparative representation of the UV signal and *z*-average diameter of NLb1 after a) 1 h incubation in serum-supplemented (S) and serum-free (M) cell culture medium, b) 4 h incubation in serum-supplemented and serum-free cell culture medium, c) 1 and 4 h of incubation in serum-free cell culture medium, d) 1 and 4 h incubation in serum-supplemented cell culture medium.

Each of the four sections (a–d) included in Figure 2 shows overlaid signals obtained from the UV detector and the *z*-average coming from the DLS operated in flow mode. It can be observed that there is no significant change in the size of NLb1 vesicles (formulation with 50 mg PEG) after their incubation in cell culture medium with and without serum for a period of 1 and 4 h. It is well known that PEGylation plays a critical role in modulating the formation and composition of the protein corona on nanoparticles by introducing a steric barrier that reduces and selectively alters protein adsorption. The extent of this modulation strongly depends on both the PEG grafting density and the molecular weight of the PEG chains. Namely, the incorporation of 50 mg of DSPE-mPEG2000 serves to introduce a PEGylated surface that effectively modulates protein corona formation upon exposure to biological media, and thereby enhancing colloidal stability and circulation time. The PEG2000 chains, with a molecular weight of approximately 2 kDa, are of sufficient length to adopt a brush-like conformation when incorporated in a higher molar ratio, limiting access of plasma proteins to the lipid surface. This high local PEG density has been shown to decrease total protein binding while selectively enriching the corona with low-affinity or dysopsonic proteins that support the “stealth” effect [26–28].

**Figure 3:**
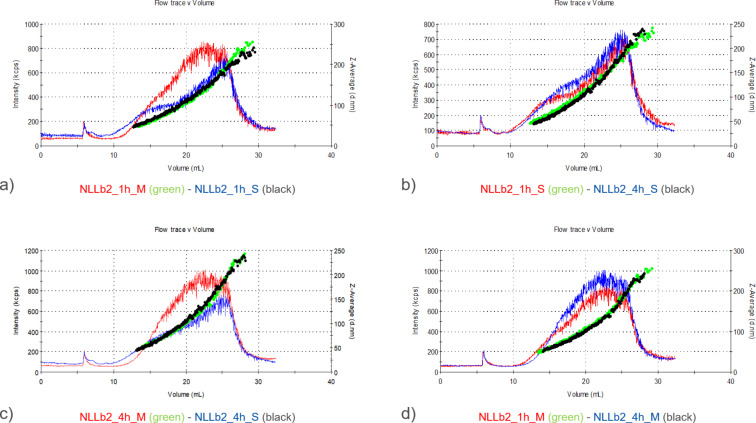
Comparative representation of the UV signal and *z*-average diameter of NLb2 after a) 1 h incubation in serum-supplemented (S) and serum-free (M) cell culture medium, b) 1 and 4 h incubation in serum-supplemented cell culture medium, c) 4 h incubation in serum-supplemented and serum-free cell culture medium, d) 1 and 4 h of incubation in serum-free cell culture medium.

During these experiments, it was also observed that the NLb2 formulation incubated for 1 h in serum-supplemented cell culture medium contained slightly smaller particles in the upper particle size range compared to that of the same sample incubated for 4 h (Figure 3b).

In addition, the measurements also showed smaller NLb2 particles in the upper size range when this formulation was incubated in serum-supplemented cell medium compared to that when it was incubated in serum-free cell medium at both time points, separately (1 and 4 h) (Figure 3a,c). In the case of particle diameter increase as a result of the PC formation, a general increase in size throughout the whole size range would be expected. In this case, the increase was only observed in the upper size range. In this direction, the obtained results can be attributed to the fact that serum proteins can also stabilize nanocarriers, thus preventing their aggregation process [29].

**Figure 4:**
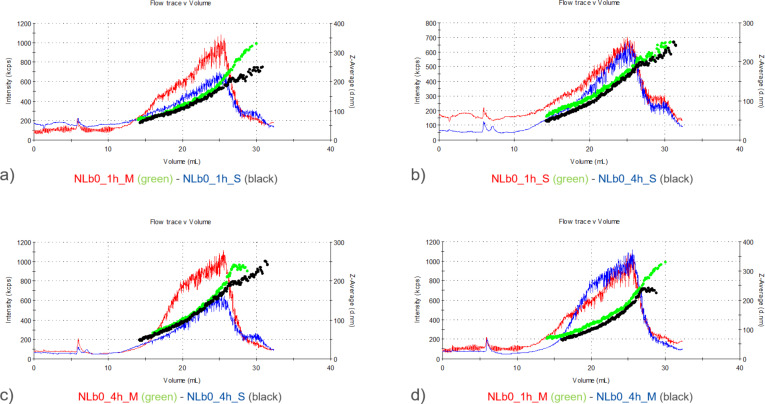
Comparative representation of the UV signal and *z*-average diameter of NLb0 after a) 1 h incubation in serum-supplemented (S) and serum-free (M) cell culture medium, b) 1 and 4 h incubation in serum-supplemented cell culture medium, c) 4 h incubation in serum-supplemented and serum-free cell culture medium, d) 1 and 4 h of incubation in serum-free cell culture medium.

Similar to NLb2, AF4 analysis showed that the particles of NLb0 (nonPEGylated formulation) were characterized by a smaller size when incubated in serum-supplemented cell culture medium compared to those incubated in serum-free medium. As already discussed, this situation can be the result of the stabilizing effect provided by the proteins present in the serum on the NLs (Figure 4a,c). However, it is interesting to note that during these studies an unexpected decrease in the size of NLs was observed after 4 h vs 1 h of incubation in serum-supplemented medium (Figure 4b), which is probably due to the fact that PC formation is a dynamic process that generally tends to evolve over time and involves many different driving forces controlled by the properties of nanosystems, proteins, and the medium itself [30]. The obtained results are in accordance to the results of the study of Miclăuş et al. [31], where it was demonstrated that the soft corona (formed at the initial time points of incubation) contains more proteins than the hard corona formed at later time intervals, resulting in a larger particle diameter at early incubation periods.

### High-resolution automated electrophoresis of adsorbed proteins onto the nanoliposomal surface

**Figure 5:**
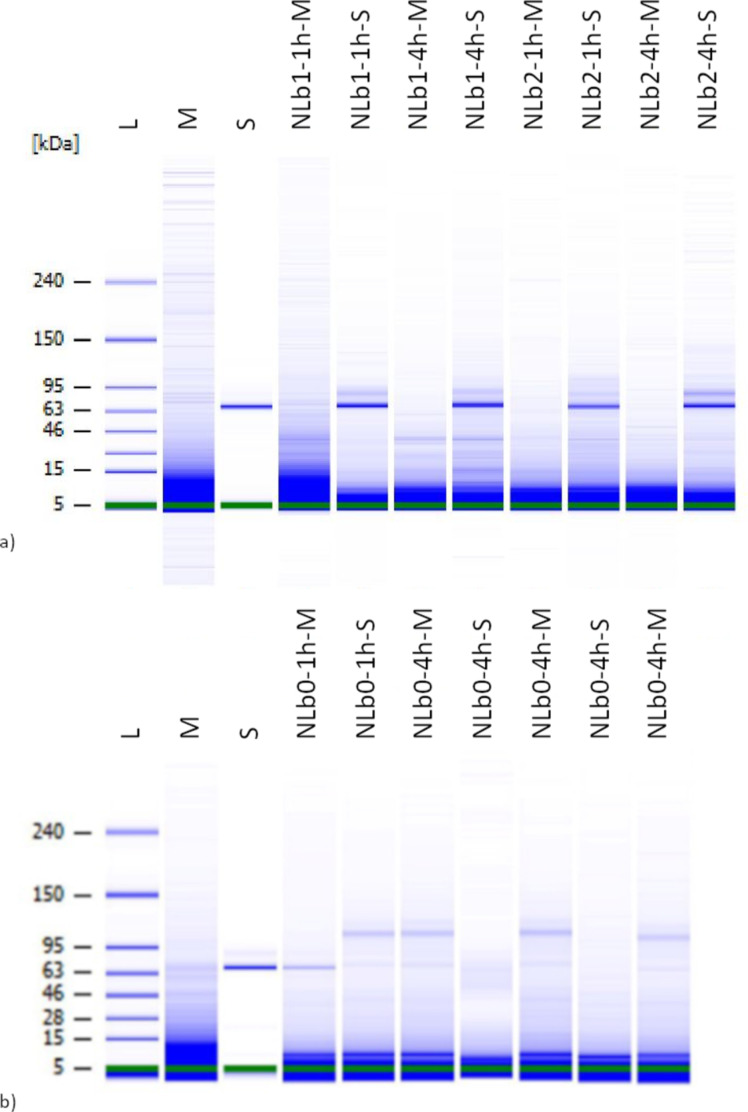
High-resolution automated electrophoresis band representation of a) NLb1 and NLb2 after 1 and 4 h incubation in serum-free cell medium (M) and serum-supplemented cell medium (S), b) NLb0 after 1 and 4 h incubation in serum-free cell medium (M) and serum-supplemented cell medium (S).

In the next step, qualitative analysis of the adsorbed serum components on the surface of the nanoformulations (NLb0, NLb1, and NLb2) was investigated (Figure 5). For this purpose, NLs were incubated in cell culture medium with and without serum (as control) for 1 and 4 h. From the graphical representations, it can be observed that the protein adsorption by NLb1 and NLb2 (Figure 5) is already expressed in the first hour of incubation, resulting in strong bands at about 60 kDA, originating from albumin, the most abundant protein in the serum. On the other hand, these bands are not so expressed in NLb0. Considering that the sensitivity of the bioanalyzer is high and it covers a wide range of concentrations, the results obtained for this formulation may be due to problems with denaturation of proteins present in the formed PC, as well as the manipulation and processing of the sample. Namely, false negatives might arise because proteins detach from the nanoparticle–corona complex under the influence of centrifugal forces. Hence, it is crucial to ascertain the optimal number of washing cycles and centrifugation duration necessary for effectively isolating a particular type of a nanosystem–corona complex from a protein-rich medium [32]. In addition, weak bands from other proteins can be observed in all three formulations, but more detailed analysis by mass spectrometry is required.

### Cell uptake assessment of nanoliposomes

As previously discussed, one of the prerequisites for achieving a therapeutic effect in the brain is the successful transport of NLs across the BBB, as well as their internalization in neurons. In this direction, after determining the safety concentration range of NLs [33], we then investigated the in vitro cell uptake of NLs by two cell lines: BBB cells (hCMEC/D3) and human neuroblastoma cells (SH-SY5Y). Quantitative uptake experiments performed on the two cells lines (hCMEC/D3 and SH-SY5Y) exposed to 5, 10, or 100 μg/mL of the NLs under investigation and at different time points (1, 2 and 4 h) are reported in Table 2.

**Table 2:** Cell uptake of NLs (μg) by hCMEC/D3 and SH-SY5Y cell lines.

hCMEC/D3 cell line				SH-SY5Y cell line		

	1 h	2 h	4 h	1 h	2 h	4 h

5 μg/mL						

NLb0	0.09 ± 0.03	0.17 ± 0.01	0.54 ± 0.09	0.19 ± 0.03	0.28 ± 0.01	0.31 ± 0.02
NLb1	0.22 ± 0.03	0.38 ± 0.03	0.55 ± 0.05	0.18 ± 0.02	0.24 ± 0.01	0.24 ± 0.01
NLb2	0.20 ± 0.02	0.23 ± 0.05	0.29 ± 0.04	0.18 ± 0.02	0.20 ± 0.01	0.21 ± 0.01

10 μg/mL						

NLb0	0.36 ± 0.01	0.59 ± 0.04	1.07 ± 0.12	0.61 ± 0.11	0.74 ± 0.04	0.85 ± 0.04
NLb1	0.46 ± 0.05	0.64 ± 0.01	1.12 ± 0.02	0.61 ± 0.06	0.66 ± 0.03	0.74 ± 0.02
NLb2	0.51 ± 0.02	0.56 ± 0.01	0.70 ± 0.12	0.51 ± 0.01	0.66 ± 0.08	0.72 ± 0.04

100 μg/mL						

NLb0	3.20 ± 0.13	3.38 ± 0.03	3.25 ± 0.06	3.67 ± 0.10	3.85 ± 0.10	4.14 ± 0.18
NLb1	2.92 ± 0.01	3.02 ± 0.07	3.06 ± 0.03	3.28 ± 0.21	3.27 ± 0.12	3.59 ± 0.07
NLb2	2.98 ± 0.07	3.03 ± 0.02	3.08 ± 0.05	3.57 ± 0.25	3.58 ± 0.10	3.81 ± 0.11

As it can be observed, there is a gradual increment of the cell uptake for all formulations with the increase of their concentrations at all time points for both cell lines analyzed, which is an expected phenomenon. Similarly, an increasing trend in the uptake can be seen with prolonged incubation time, except for the highest concentration tested (100 μg/mL), for which differences were found between the two cell lines. At this concentration, the uptake measured of all NL formulations was around 3 µg at all incubation times for the hCMEC/D3 cell line, whereas for SH-SY5Y it varied from 3.28 ± 0.21 to 4.14 ± 0.19 μg at 1 and 4 h, respectively. This situation may be due to the fact that the internalization of nanoliposomes can take place through several energy-dependent endocytic pathways (i.e., phagocytosis, clathrin-mediated endocytosis, caveolin-mediated endocytosis, clathrin/caveolae-independent endocytosis, and micropinocytosis), as well as through passive transport or diffusion which is an uncompetitive movement of the nanosystems, either directly through membrane phospholipids (simple diffusion) or in combination with membrane proteins (facilitated diffusion) [8,34]. Therefore, endocytosis is a process that occurs through membrane–particle adhesion followed by elastic deformation of the cell membrane and receptor diffusion to the surface of the membrane, processes highly dependent on the physicochemical properties of the NLs, as well as on their concentration and exposure time [34]. As no significant increase of uptake was found for the highest concentration tested for all three NLs and for both cell lines, it is expected that at 1 h, hCMEC/D3 and SH-SY5Y cells have already reached their maximal endocytic potential, which is probably due to the saturation of uptake mechanisms leading to limited internalization [35–37]. Additionally, as previously elaborated, the increased uptake by increasing the incubation time observed at 5 and 10 µg/mL for all NLs investigated confirms the lack of a saturable transport process [38].

In order to investigate the influence of the possible independent factors (experimental conditions – time of incubation/NLs concentration, type of formulation/amount of PEG) on the quantitative uptake of the NLs in the specified cell line cultures, a multivariate statistical analysis was performed. The correlation coefficients obtained from the initial model comprising all internalization data (normalized uptake – %) were low, but it was observed that the cell type predominantly affects the scores of the individual points, which is why PLS-DA was decided to be performed. In continuation to the aforementioned discussed, for the internalization kinetic experiments, separate multivariate statistical models were done for each cell culture. The internalization model in hCMEC/D3 also confirmed that the sample concentration and the exposure time were dominant factors on the percentage of NLs taken up (Supporting Information File 1, Figure S3a–c). According to the VIP plot, the formulation type (i.e., the amount of PEG on the surface) also had a significant effect on the uptake (Supporting Information File 1, Figure S3d). The model of kinetic experiments on the SH-SY5Y cell line presents a similar behavior to the previous model, where concentration and exposure time were the dominant factors affecting uptake, while the amount of PEG on the surface has a smaller but distinctive influence (Supporting Information File 1, Figure S4a–d).

As it can be seen from Table 2, the uptake of NLs by hCMEC/D3 after 4 h of incubation is the highest for the formulation with the highest amount of PEG on the surface – NLb1 (0.55 ± 0.05 μg and 1.12 ± 0.02 μg, at 5 and 10 μg/mL treatment concentrations, respectively), followed by the formulation with no PEG on the surface – NLb0 (0.54 ± 0.09 and 1.07 ± 0.12 μg, at 5 and 10 μg/mL treatment concentrations, respectively). The lowest rate of internalization was observed in the formulation with 5 mg of PEG on the surface – NLb2 (0.29 ± 0.04 and 0.70 ± 0.12 μg, at 5 and 10 μg/mL treatment concentrations, respectively). These differences in the cell uptake between the three formulations can be attributed to the NL stability primarily governed by the dynamic process of PC formation. Namely, this phenomenon can significantly influence and dictate the cell recognition, cell membrane adhesion and interactions, the internalization mechanisms, as well as the intracellular trafficking of nanosystems since it gives them new biological identity [39]. The impact of PC on particle–cell interactions varies based on particle properties and cellular components, as well as the nature of the cell culture medium and its components such as serum as well as growth factors, dyes, and antibiotics [40–42]. Apart from the already reported greatest stability in cell culture medium over time (by AF4 analysis), the highest cell uptake NLb1 can be also attributed to its *z*-potential which is less negative (−15 mV), compared to the other two formulations, since less negatively or positively charged nanocarriers would be expected to be more efficient in crossing the negatively charged BBB [43]. In addition to this, several research groups have shown that the PEG surface density and conformation also play a key role and improve the diffusion and transport of different types of nanosystems across endothelial barriers, particularly the BBB, and consequently, their brain distribution. Taking into consideration that NLb1 exhibits high amount of PEG, the density of the chains on the liposome surface is expected to be increased and be characterized with a“dense brush” conformation as steric hindrances restrict movement and self-coiling of the grafted polymer [44]. This fact is supported by the literature data that confirms that nanosystems characterized by “dense brush” PEG coating can permeate the BBB and more efficiently accumulate in the brain parenchyma ex vivo in comparison with uncoated PEG [45].

From Table 2, it can also be observed that during 1 h of incubation intervals the cell uptake amount of NLb0 is lower compared to that of NLb2, at both treating concentrations (5 and 10 µg/mL). However, the opposite case is noticed over 4 h of incubation, where the quantitative cellular uptake of NLb0 was approximately 1.5 fold higher than that of the uncoated formulation. The main reason for this can be the fact that in our previous stability studies conducted by AF-4 analysis, it was shown that serum proteins present in the cell culture medium stabilize NLb0 in terms of preventing the process of aggregation. Additionally, unlike for NLb2, there was a decrease in the average diameter over the incubation time of 4 h, which is probably due to the dynamic process of PC formation. Hence, these results only confirm the statement regarding the opposite dependence between the particle size and hCMEC/D3 liposomal uptake and adhesion as well as the alterations on the internalization promoted by the adsorbed serum proteins onto the surface of NLs [46].

On the other hand, NLb2, despite the low amount of PEG on its surface, also showed saturable uptake within the first hour (0.20 ± 0.02 and 0.51 ± 0.02 μg, at 5 and 10 μg/mL treating concentrations, respectively), since the difference in the amount of internalized particles of NLb2 in the later incubation times (0.29 ± 0.04 and 0.70 ± 0.12 μg after 4 h, at 5 and 10 μg/mL treating concentrations, respectively) tended to fade out and become significantly lower compared to the other two formulations. These results additionally confirm the limited capacity of NLb2 to accumulate intracellularly, and is also indicative of an equilibrium between endocytosis and exocytosis [47]. Additionally, the literature data suggests that PEG-coated particles with a surface charge between −20 and −40 mV are not capable to cross the BBB probably due to the insufficient dense coating of PEG ([45]). This is in accordance with our results since we can conclude that hydrophilicity as well as surface charge can significantly affect the nanosystem delivery to the BBB, and thus brain tissues.

When it comes to the cellular uptake of the NLs by the SH-SY5Y cell line, there is a different trend of quantitative internalization among the formulations. Namely, the formulation characterized by the highest cellular uptake after 4 h is the nonPEGylated NLb0 (0.31 ± 0.02 and 0.85 ± 0.04 μg, at 5 and 10 μg/mL treating concentrations, respectively), followed by NLb1 (0.24 ± 0.01 and 0.74 ± 0.02, at 5 and 10 μg/mL treating concentrations, respectively). The same situation as in hCMEC/D3, NLb2 was observed to have the lowest cellular uptake with 0.21 ± 0.01 and 0.72 ± 0.04 μg, at 5 and 10 μg/mL treating concentrations, respectively. The obtained results are in accordance with the literature data suggesting that nonPEGylated liposomes are prone to more efficient uptake by neuroblastoma cells. This outcome is probably due to the fact that PEG chains hinder the interactions of the liposomes with different membrane structures of this type of cells, thus resulting with poor intracellular transport [48]. On the other hand, neurons exhibit membranes which are unique in its composition being highly enriched in lipids, in particular cholesterol, which plays a key role in regulating the membrane structure, fluidity, and permeability as well as multiple aspects of the synaptic transmission [49]. In pure human SH-SY5Y cell cultures, the glia-derived cholesterol is nonexisting, and addition of cholesterol is needed in order to achieve conditions resembling normal neuronal environment with surrounding glial cells, as well as to promote the process of the SH-SY5Y neuroblastoma cell differentiation into a neuronal cell type [50]. Up to date, several findings reported the clear preference of SH-SY5Y neurons for cholesterol-containing liposomes. Namely, Lee et al. [51] reported that the addition of cholesterol into the liposomal formulation resulted in a 11-fold enhanced uptake by this cell culture line, implying on the fact that the composition of NLs significantly affects their uptake by neuronal cells and are avidly taken up by the addition of cholesterol. In this sense, since all three nanoformulations contain cholesterol into their lipid bilayer, the lower uptake of the PEGylated liposomes (NLb1 and NLb2) may be a result of the steric effect of the PEG chains onto the surface, which probably act as a barrier and prevent the access of cholesterol to the cellular structures. It is also important to mention that PEGylation can prevent or reduce but does not totally exclude the protein binding to the NL surface. Additionally, excessive PEGylation may contribute to less efficient binding with protein targets that would work as ligands for receptor-mediated transport and delivery, finally resulting in partial inhibition and reduction of cellular uptake ([24]).

Taken into consideration all aforementioned, it can be summarized that cellular transport and internalization are influenced by numerous features such as NL composition (especially the amount of PEG), physicochemical properties of NLs, the experimental conditions (concentration and incubation time), as well as the composition of the cell medium and structural characteristics of the cell culture lines.

### Cell uptake assessment of nanoliposomes in the presence of transport pathway inhibitors

In order to have insights into the mechanism of internalization of the NLs, as well as to better understand and correlate with previously presented quantitative results for cell internalization at 37 °C, uptake experiments in the presence of specific inhibitors of endocytotic pathways were performed. In this sense, the cell culture lines (hCMEC/D3 and SH-SY5Y) were pretreated (40 min) with chlorpromazine or indomethacin as specific inhibitors of clathrin- and caveolin-mediated endocytosis. In addition, uptake experiments at 4 °C were conducted, when it is supposed that all ATP-dependent transport mechanisms are blocked (Supporting Information File 1, Table S1). The fluorescence of cells incubated with NLs at 37 °C was considered as 100%, while the fluorescence after incubation in the presence of inhibitors was expressed as a relative percentage compared to the cells without inhibitors.

The statistical analysis of the obtained results for hCMEC/D3 cells (Supporting Information File 1, Figure S5a–c) clearly shows the concentration and endocytosis inhibitors having a significant effect, while lowering the temperature of the experiment (total energy metabolism) had no significant effect on the total uptake. In addition, the type of formulation (i.e., the amount of PEG on the NL surface) also affects the uptake, as seen on the VIP plot which provides an overall representation of the effect of the independent variables (Supporting Information File 1, Figure S5d). The SH-SY5Y uptake pattern showed a different trend of the influence of the independent variables (Supporting Information File 1, Figure S6a–c). According to the VIP plot (Supporting Information File 1, Figure S6d), it can be concluded that the concentration of the sample and also the temperature of the experiment are dominant factors affecting the uptake. Similar to hCMEC/D3, the type of formulation, or more precisely, the PEG amount, also demonstrated a significant effect on the uptake under varying experimental conditions.

**Figure 6:**
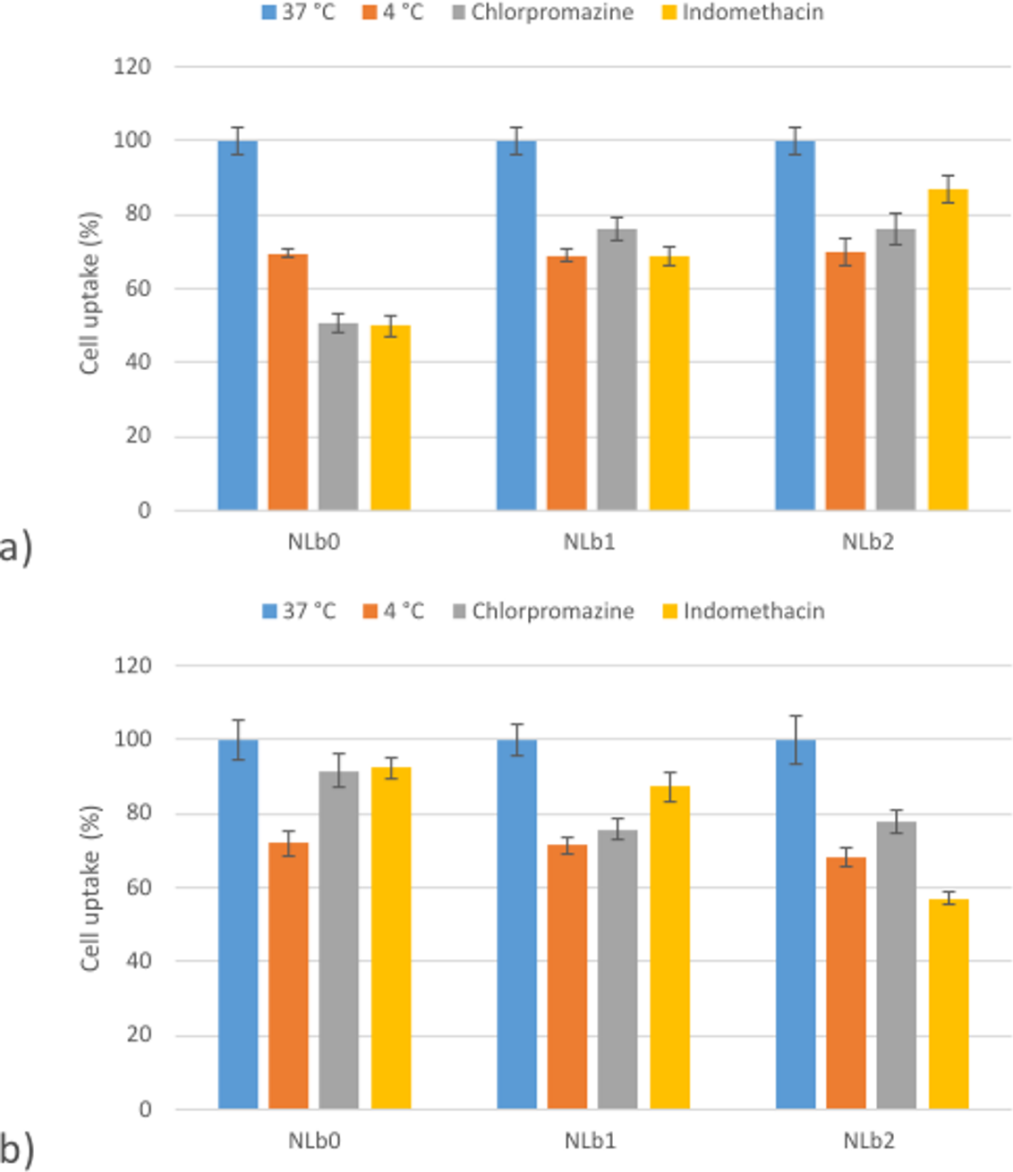
Cell uptake of NLs (10 μg/mL) in a) hCMEC/D3 cells and b) SH-SY5Y after a 2 h incubation at 4 °C with chlorpromazine or indomethacin.

From Figure 6, it can be observed that incubation at 4 °C induces cell metabolic inhibition, resulting (for the concentration of 10 μg/mL) in a reduction of ≈30% of the uptake of all formulations in both cell lines compared to that of the experiments performed at 37 °C. This indicates that energy-dependent endocytosis is included in the NL uptake along with physical adhesion or passive diffusion [16].

In order to investigate the mechanism of endocytosis, cells were also treated with chlorpromazine which is known to inhibit AP2, one of the key adaptor proteins in clathrin-mediated endocytosis. It is also involved in clathrin accumulation in late endosomes, thereby inhibiting coated-pit endocytosis. Figure 6 shows a decrease in the uptake of NLb1 and NLb2 by ≈25% in both cell lines compared to the control at 37 °C, referring to the fact that clathrin-mediated endocytosis may be involved as one of the predominant internalization pathways for the uptake of NPs ≈120 nm, given the size of clathrin-coated pits [16,52–53]. Additionally, the performed experiments resulted in significant reduction of the uptake of NLb0 in hCMEC/D3 (50.59 ± 2.65%), whereas only a slight decrease was observed in SH-SY5Y (93.78 ± 4.58%). This could be due to different structural specificities and distinct cell surface properties as well as the specific PC formed onto the surface of NLs after incubation with cell culture medium [54]. On the other hand, it should be taken into consideration that when attempting to block a certain transport pathway, different types of cells usually adapt via activation of alternative mechanisms as well as overcompensation for the blocked function or receptor [52]. This statement can further explain the heterogeneous results obtained for the inhibition of caveolin-mediated endocytosis with indomethacin between different formulations in different cell lines (49.96 ± 2.95–87.10 ± 3.56% and 57.17 ± 1.56–92.38 ± 2.65%, for hCMEC/D3 and SH-SY5Y, respectively). The surface properties of the nanosystems such as PEGylation can also affect the cell uptake/adhesion since the conformation of PEG chains and also the aggregation of PEG polymers in the contact region between a PEGylated liposome and the membrane can influence the membrane-wrapping process of PEGylated liposomes during endocytosis [55]. Therefore, different energy-dependent and independent pathways are probably included in the dictation of NL transport across the BBB and neurons.

### Cell uptake experiments on co-cultured hCMEC/D3 and SH-SY5Y cells

Several studies have reported the internalization and uptake of NLs by different types of neuronal and BBB cell culture lines, individually. In this sense, detailed experiments were conducted under different experimental conditions on the two cell culture lines, hCMEC/D3 and SH-SY5Y, in order to determine the quantitative cell uptake and predict the internalization mechanism of the investigated NLs. However, despite the confirmed uptake of SH-SY5Y neuroblastoma cells after direct exposure to NLs presented earlier, it is not certain whether the results would be consistent and the obtained effects would be replicated in vivo, where the ability of nanocarriers to serve as platforms for active components intended for CNS treatment is limited due to the primary challenge of permeating across the BBB [56]. Another point that should be taken into consideration is the fact that the information regarding the fate of the nanosystems in pericytes, astrocytes, or neurons after having crossed the BBB is quite limited [57].

Recent studies have highlighted the pivotal role of co-culture models in advancing in vitro neurotoxicity research. These models have significantly contributed to bridging the gap in accurately replicating the human BBB phenotype. This is crucial for conducting permeability studies, assessing neurotoxicity, and investigating aspects related to neurodegenerative diseases [58]. The findings from the study of Freese et al. [59] illustrates the effectiveness of a new hCMCEC/D3 – SH-SY5Y bioassay in vitro system which can predict drug penetration across the BBB, particularly for drugs relevant to Alzheimer's disease (AD) therapy. The same in vitro model with slight modifications was used by Mursaleen et al. [60] in order to demonstrate that micellar nanocarriers loaded with hydroxytyrosol effectively crossed the BBB in vitro without inducing cytotoxicity. Moreover, these nanocarriers protected neuronal SH-SY5Y cells against rotenone-induced oxidative stress, as assessed by mitochondrial hydroxyl levels. However, it is important to note that in this study, the result evaluation was done based on the biological activity of the drug, not through the measurement of the permeation and uptake of micelle carriers by brain cells.

When it comes to permeability studies of lipid nanoparticles with different surface characteristics across hCMEC/D3 and their subsequent uptake in SH-SY5Y, the literature is limited and generally focused on research involving ligand-functionalized lipid nanosystems. In this context, one of the few studies available is the evaluation of the efficacy of apolipoprotein-E- (APOE) targeting nanoparticles for delivering donepezil across the BBB. The results underscored the effective permeability of targeting nanoparticles across the BBB, and the findings indicated that nanoparticles equipped with APOE-targeting ligand demonstrated higher cellular uptake compared to that of the nonfunctionalized ones [61].

For this reason, human-derived brain endothelial cells, hCMEC/D3, were cultured on the apical side of permeable Transwell inserts, while monolayers of SH-SY5Y neuroblastoma cells were seeded onto the basal side of 12-well plate chambers. After hCMEC/D3 reached a TEER value >230 Ω and the confluence of SH-SY5Y was >85%, both cell culture lines were combined and transport studies of the three NL formulations were performed.

The results from the NL cellular uptake into neuronal cells after crossing the blood-brain barrier in vitro in our study suggest that the nonPEGylated formulation (NLb0) is more internalized (27.54 ± 2.93%), followed by NLb2 (26.46 ± 1.87%) and by the formulation with the highest amount of PEG onto its surface – NLb1 (25.17 ± 1.74%). This implies the successful transport of these liposomal nanocarriers across the BBB model and the consequent uptake of the particles by the neuronal cells [60]. It was also noticed that the quantitative uptake trend by SH-SY5Y in combination with hCMEC/D3 for the three different formulations is in good relation to the experiments on the single cell line (33.27 ± 1.95, 25.17 ± 2.65, and 26.46 ± 1.54% for NLb0, NLb1 and NLb2, respectively), which could be attributed to the physicochemical properties of NLs and physiological factors affecting their internalization as well as the morphological properties of SH-SY5Y already discussed. In summary, while the results from each individual cell line experiments were consistent with known PEGylation effects, the co-culture Transwell model provided a more integrated perspective on the dynamic interplay between surface modification and cellular uptake, simulating a more physiologically relevant barrier-crossing scenario.

### Internalization studies

**Figure 7:**
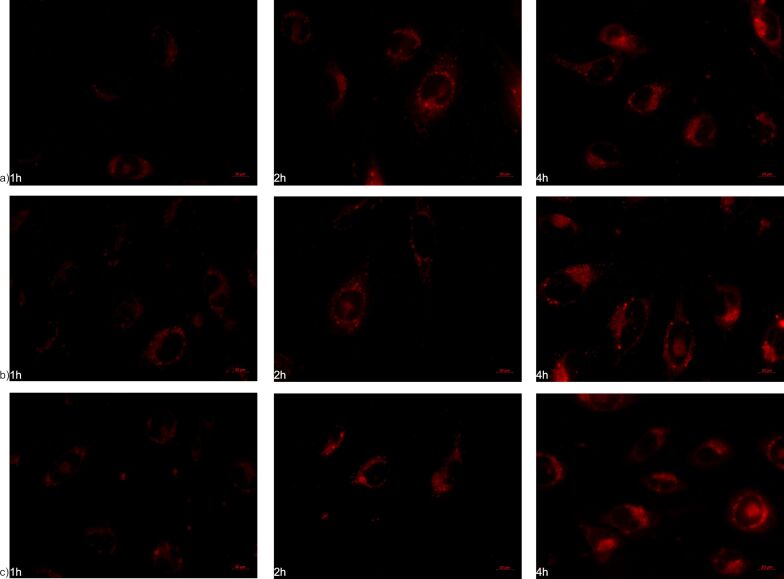
Fluorescent microscopy (Nile-red channel used) showing internalization in live hCMEC/D3 cells of a) NLb0, b) NLb1, and c) NLb2.

**Figure 8:**
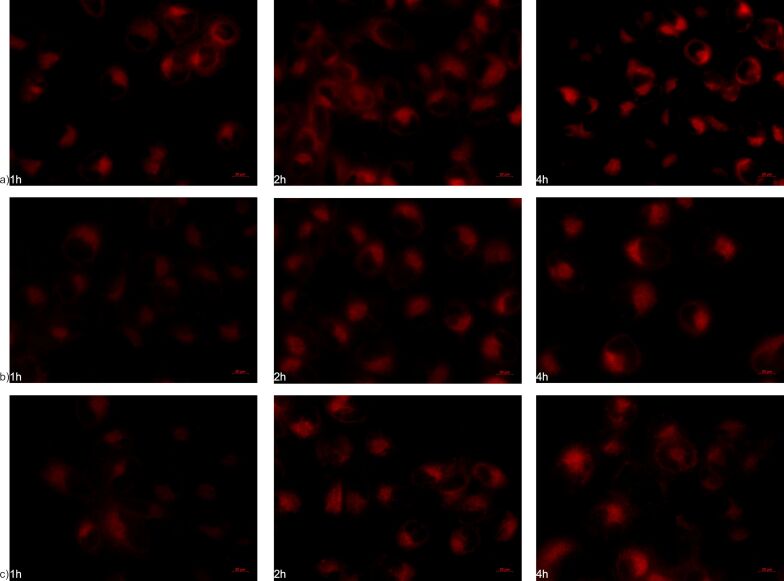
Fluorescent microscopy (Nile-red channel used) showing internalization in SH-SY5Y cells of a) NLb0, b) NLb1, and c) NLb2.

To further verify the aforementioned results, the internalization of NLs by hCMEC/D3 and SH-SY5Y cell lines was investigated using fluorescent live-cell imaging and confocal microscopy. Figure 7a–c and Figure 8a–c show images obtained by fluorescent microscopy of NLs incubated in both cell lines for 1, 2, and 4 h. From the microscopic images, the time-dependent internalization of all three formulations can be observed in both cell lines, where higher amounts of internalized nanoliposomal vesicles were noted in later time intervals. Additionally, from the fluorescence intensity, it can be seen that in the cells of the blood-brain barrier, the largest amount of internalized vesicles is attributed to NLb1, followed by NLb0 and NLb2, respectively, whereas in neuroblastoma cells, NLb0 exhibits the highest percentage of uptake, which is consistent with previous studies on the quantitative uptake at 37 °C.

**Figure 9:**
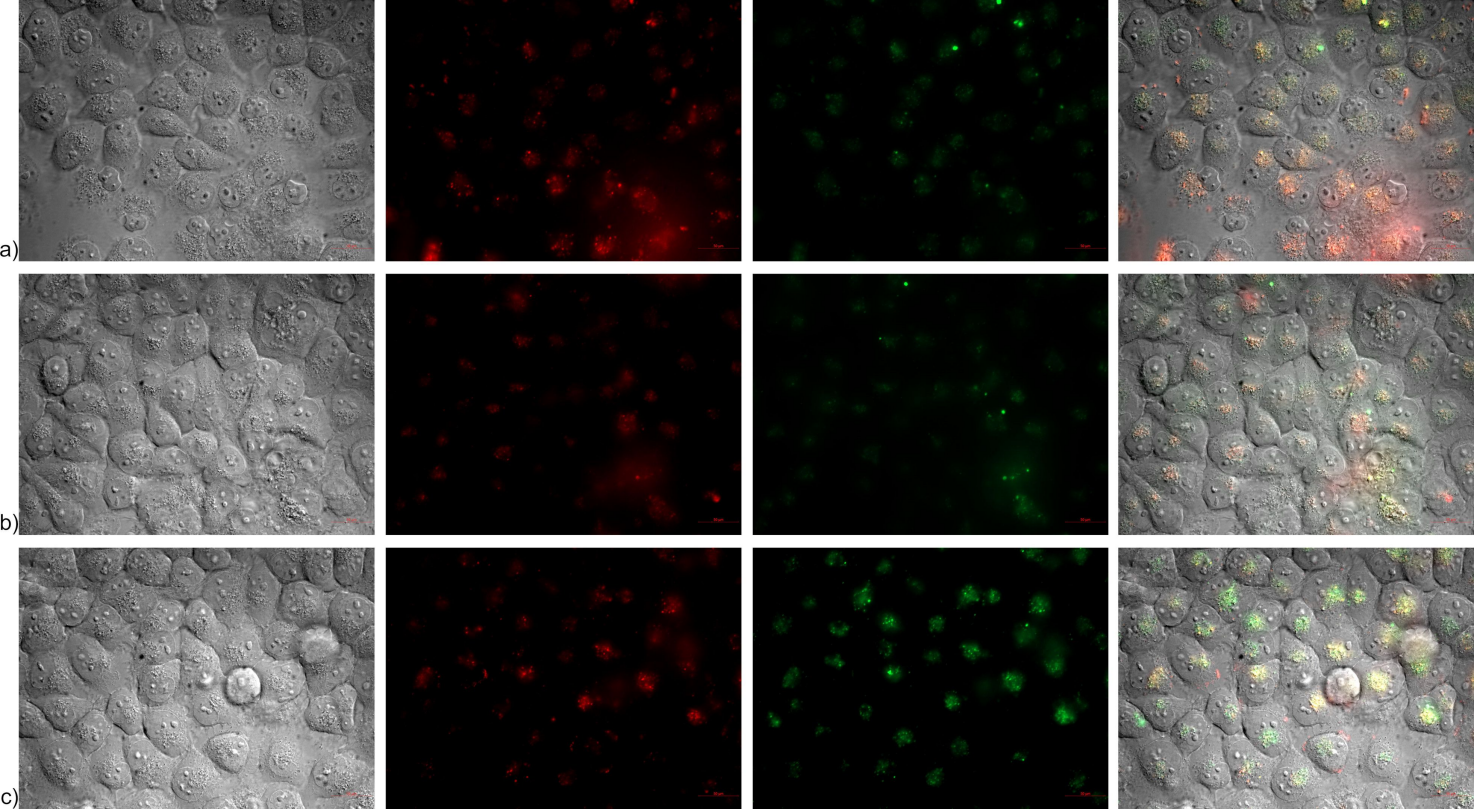
Representation of a) NLb0, b) NLb1, and c) NLb2 in live SH-SY5Y cells (4 h) by fluorescent microscopy. Left image – phase contrast; second image – red fluorescence by NLs marked with Dil red; third image – green fluorescence by endosomes marked with pHrodo Green dextran conjugate, right image – superimposed image.

Many research studies have demonstrated that liposomes tend to follow an endocytic mechanism of cellular internalization. Therefore, it was also important to visualize the NL internalization pathway and confirm their co-localization in endosomes. In this direction, NLs were incubated for 4 h in the presence of a dye that signals endocytosis, (i.e., in an acidic environment (endosomes and lysosomes) it emits green fluorescence). From the presented images on Figure 9a–c it could be seen and confirmed that all formulations of NLs have been internalized and co-localized in the endosomal compartments in SH-SY5Y cells.

**Figure 10:**
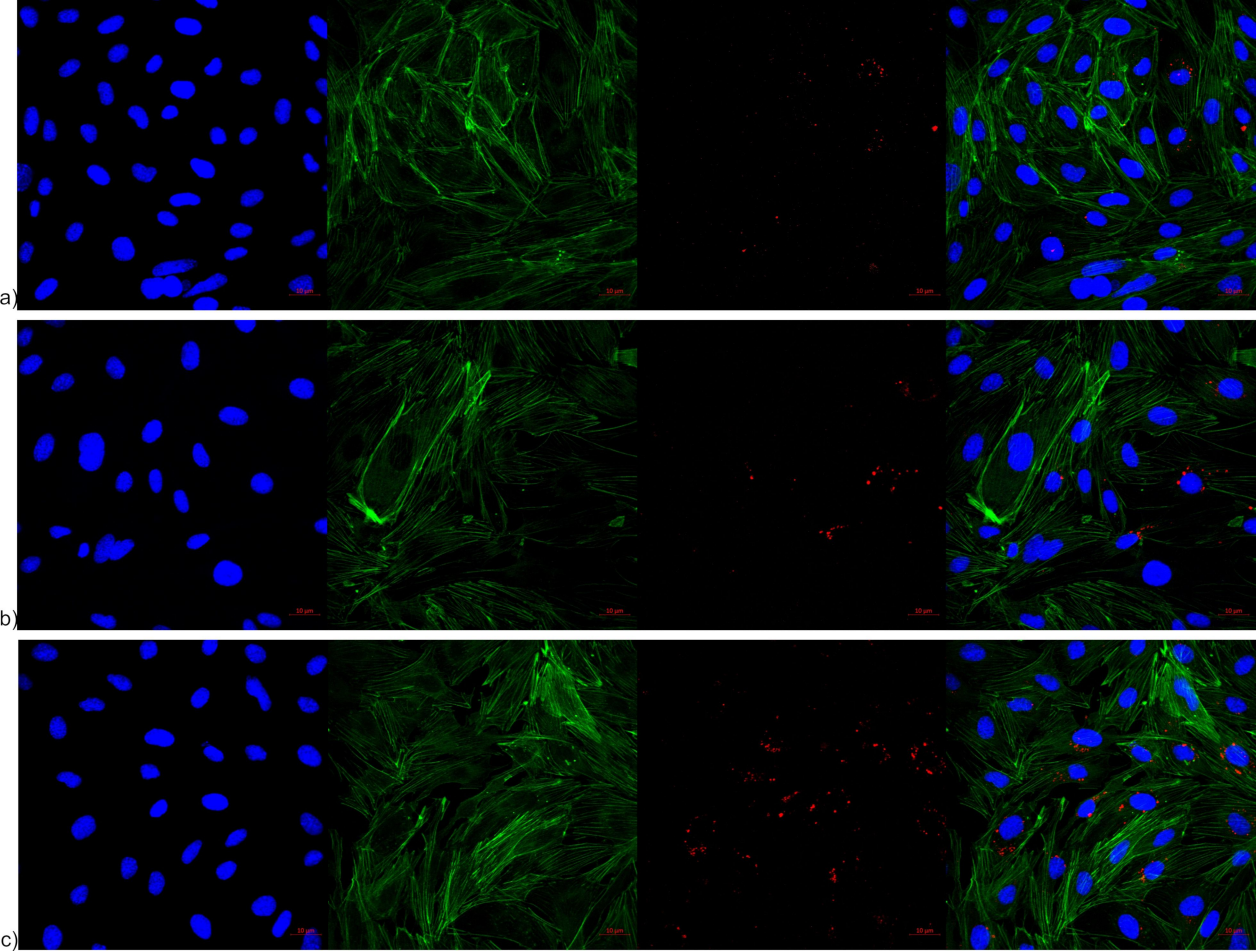
Confocal microscopy showing internalization and distribution of a) NLb0, b) NLb1, and c) NLb2 in hCMEC/D3 cells. Left image – blue channel – nucleus counterstained with Hoechst fluorescent stain and DAPI excited at 405 nm and detected by a band-pass filter (BP 420/480 nm). Second image – green channel – actin cytoskeleton stained with Alexa Fluor 488 Phalloidin excited at 488 nm and detected by a band-pass filter (BP 505/550 nm). Third image – red channel – Dil-labelled samples detected at a 549 nm excitation wavelength by a long-pass filter (LP 560 nm). Right image – superimposed micrograph.

From the images obtained by confocal microscopy, the internalization of NLs can be confirmed in hCMEC/D3 cells. Literature data suggests that lipid NLs have the ability to cross the BBB despite its highly restrictive nature, and moreover, to deliver the encapsulated drugs in different cell compartments [62]. When it comes to nanoliposomes, as it could be seen from the presented images in Figure 10a–c, they show a tendency to accumulate around the perinuclear area [63]. Regarding the intracellular localization of NLs, the obtained results showed that there is no difference in the cell distribution of the different NL formulations, or more precisely, the presence and the amount of PEG on their surface did not influence the intracellular NL co-localization (Figure 10).

## Conclusion

In this work, three different nanoliposomal formulations with different PEG amounts on the surface were prepared and appropriately characterized in a biorelevant manner. The results from the stability studies of the tested formulations confirmed that after incubation in cell culture medium there were no changes in the mean *z*-size of the sample with the highest amount of PEG (NLb1), which also confirmed the stability of this formulation. Additionally, serum proteins were found to likely stabilize the PEG-free formulation (NLb0) in terms of preventing the aggregation process. Furthermore, by electrophoresis experiments, it was evident that protein corona was formed within the first hour of incubation in the serum-supplemented culture medium, and the protein that was adsorbed in the largest percentage on the surface of NLs was albumin. However, in future studies, MS-based proteomic profiling will be essential to complement this work and elucidate the specific proteins involved, which may further explain the observed uptake patterns at a molecular level. Statistical analysis performed on the cell uptake pattern showed that NL concentration and incubation time play a key role on the percentage of internalized NLs. Furthermore, the highest uptake by hCMEC/D3 cells was obtained for the formulation with the highest amount of PEG on the surface (NLb1). A different situation was observed for the cellular uptake by SH-SY5Y cells, where the PEG-free formulation (NLb0) had the most successful internalization. When it comes to the mechanism of cellular internalization, all nanovesicle samples were characterized by energy-dependent endocytic transport and passive diffusion. The transport studies on the combined hCMEC/D3/SH-SY5Y cell line confirmed the successful transport of the nanoformulations across the BBB and their subsequent uptake by the neuroblastoma cells. The obtained micrographs from the fluorescent microscopy on live cells and the confocal microscopy gave insight into the successful internalization of the NLs in the BBB and neuroblastoma cells, revealing that the co-localization of the NLs was in the perinuclear cell regions. From the aforementioned, it can be concluded that all properties and performances of the designed NLs are in favor of an efficient brain delivery, and hence their potential for treatment of different CNS diseases.

## Supporting Information

Figures for light scattering signal at 90° / the related geometrical radii and DLS in-line measurements (Figures S1 and S2); Statistical analysis of the obtained results for cell uptake of NLs performed by Simca 14.1 (Sartorius Stedim Biotech, Germany) with included score scatter plots and VIP score for discriminative analysis of the experimental factors affecting NLs uptake in hCMEC/D3 and SH-SY5Y, for each cell uptake experiment, separately (Figures S3, S4, S5, S6); Table with results for cell uptake of NLs (μg) by hCMEC/D3 and SH-SY5Y in the presence of transport inhibitors (Table S1).

File 1 Additional figures and tables.

## Data Availability

Data generated and analyzed during this study is available from the corresponding author upon reasonable request.
