# Supplementary material for: Influence of surface characteristics on the in vitro stability and cell uptake of nanoliposomes for brain delivery
Source: Beilstein J Nanotechnol. 2026 Jan 13;17:139–58. doi: 10.3762/bjnano.17.9 (PMC12816991; doi:10.3762/bjnano.17.9)
Supplement: File 1 — Additional figures and tables. [file Beilstein_J_Nanotechnol-17-139-s001.pdf]

BEILSTEIN JOURNAL OF NANOTECHNOLOGY

Supporting Information

for

Influence of surface characteristics on the in vitro stability and cell uptake of nanoliposomes for brain delivery

Dushko Shalabalija, Ljubica Mihailova, Nikola Geskovski, Andreas Zimmer, Otmar Geiss, Sabrina Gioria, Diletta Scaccabarozzi and Marija Glavas Dodov

Beilstein J. Nanotechnol. **2026,** *17,* 139–158. doi:10.3762/bjnano.17.9

Additional figures and tables

License and Terms: This is a supporting information file under the terms of the Creative Commons Attribution License (https://creativecommons.org/licenses/by/4.0). Please note that the reuse, redistribution and reproduction in particular requires that the author(s) and source are credited and that individual graphics may be subject to special legal provisions.
The license is subject to the *Beilstein Journal of Nanotechnology* terms and conditions: (https://www.beilstein-journals.org/bjnano/terms)

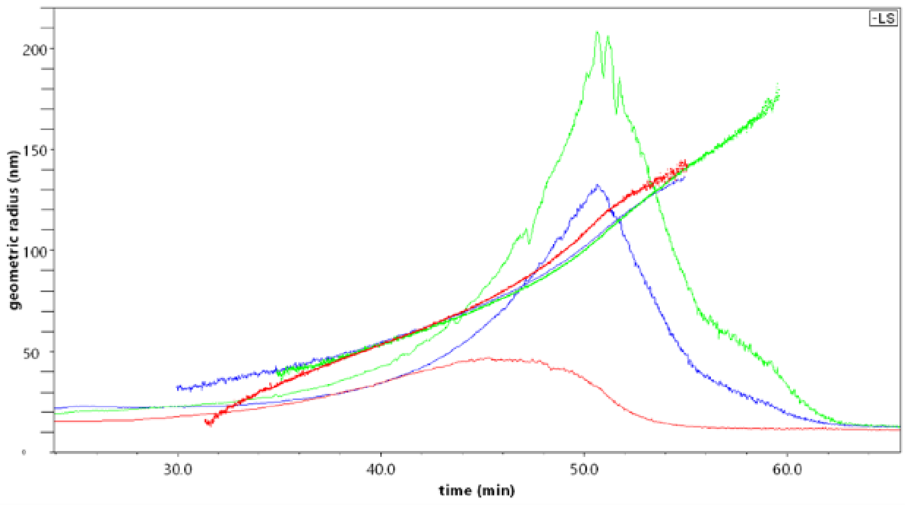

Figure S1: Light scattering signal (90°) and geometric radius [nm] of AF4-fractionated native formulations (green line: NLb0, red line: NLb1; blue line: NLb2).

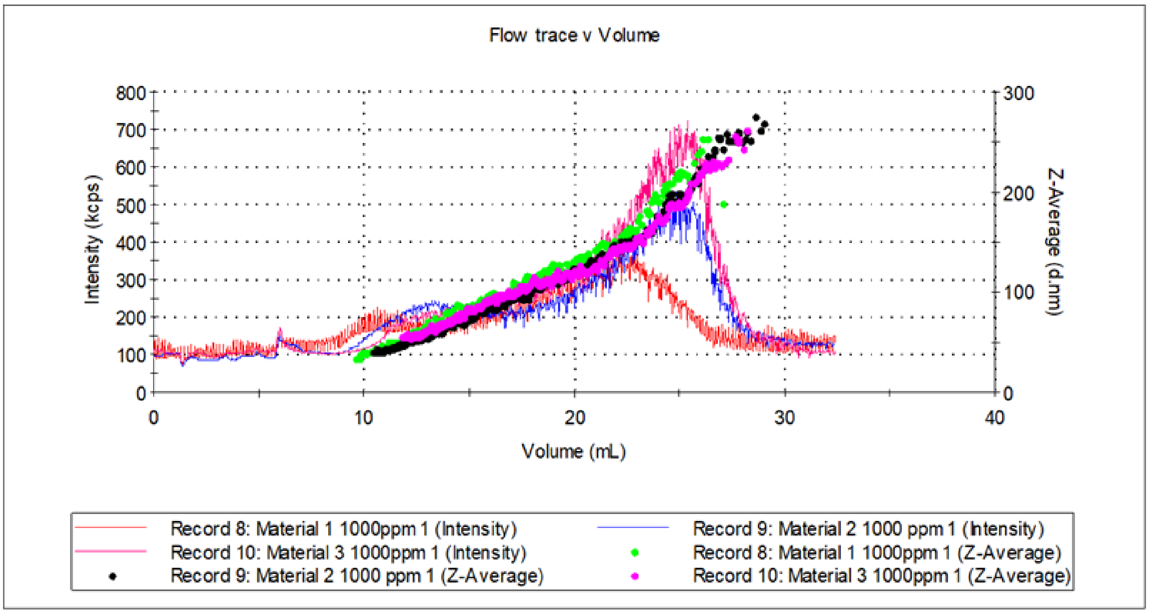

Figure S2: UV signal and *z*-average diameter of the native formulations fractionated with AF4 (Material 1 – NLb1, Material 2 – NLb2, Material 3 – NLb0).

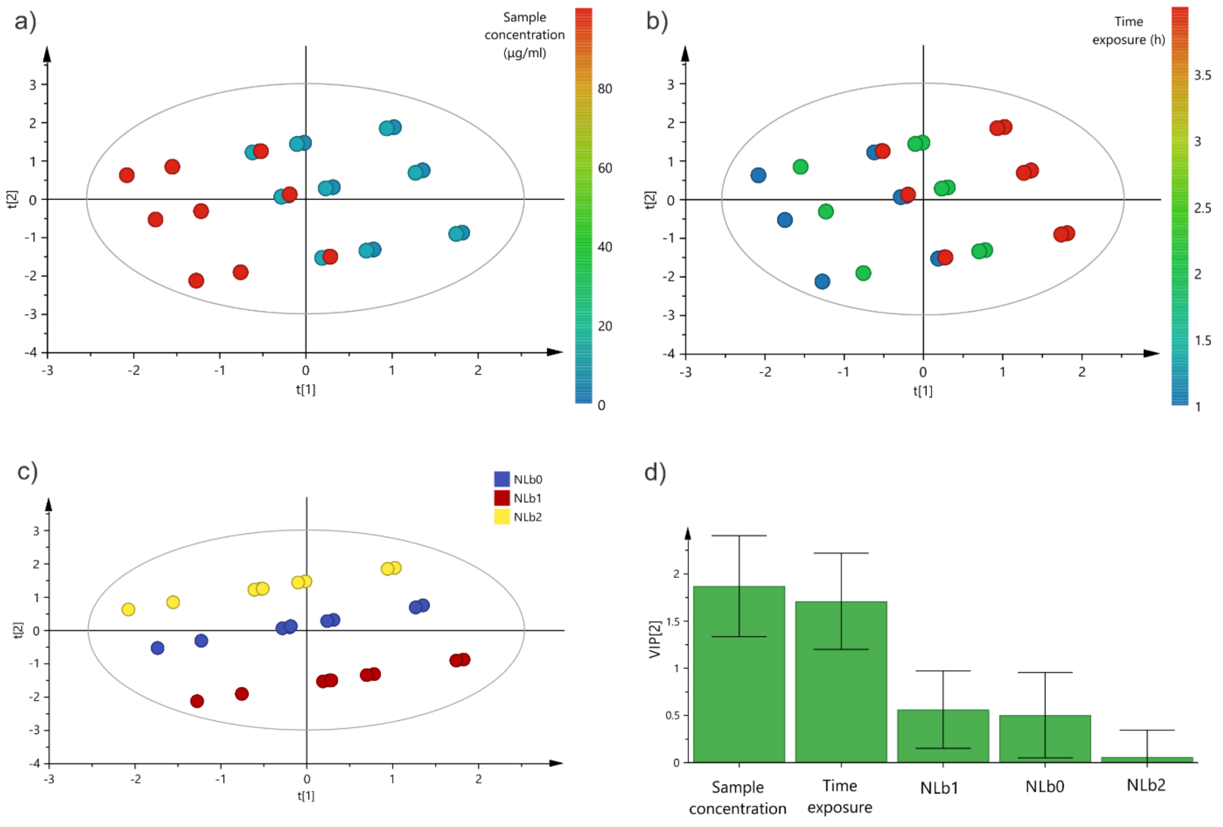

Figure S3: Score scatter plot colored by a) sample concentration, b) time exposure, c) formulation type, and d) VIP score for discriminative analysis of the factors affecting NL uptake in hCMEC/D3.

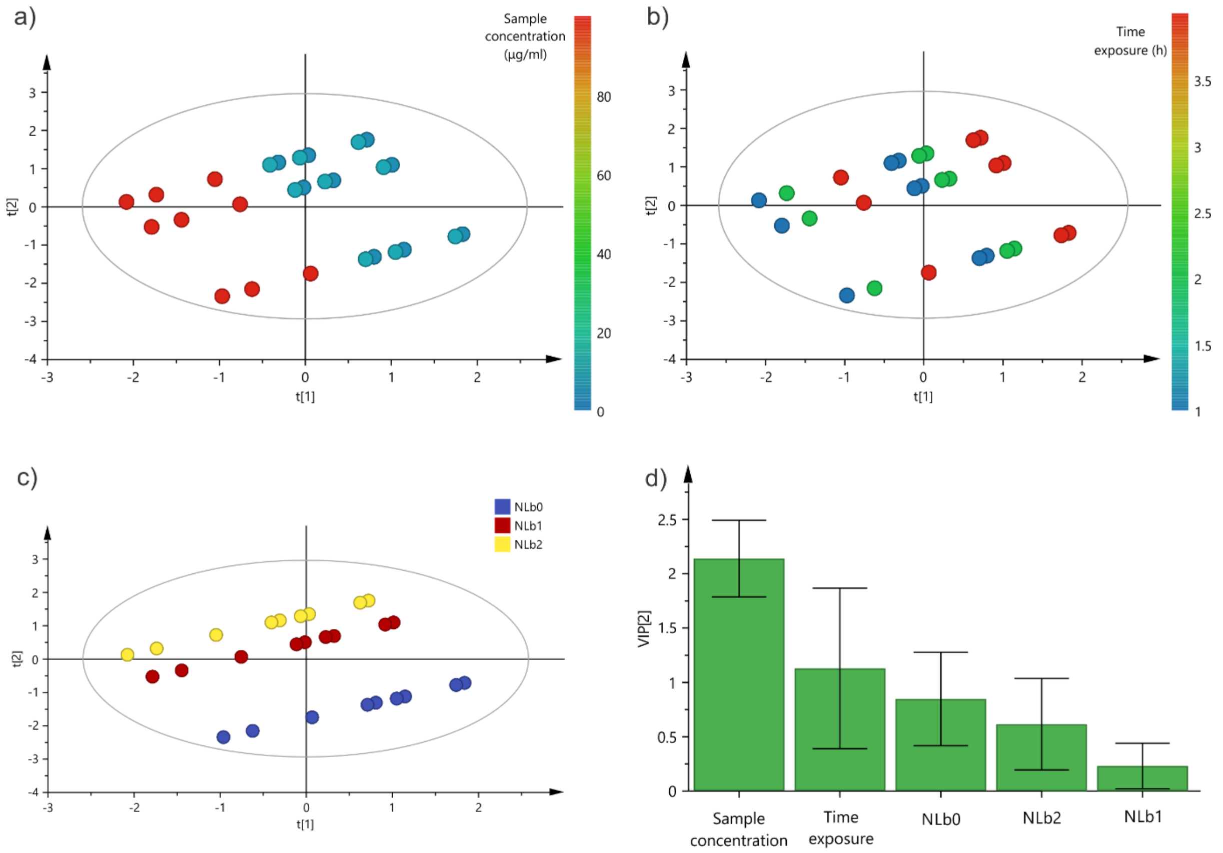

Figure S4: Score scatter plot colored by a) sample concentration, b) time exposure, c) formulation type, and d) VIP score for discriminative analysis of the factors affecting NL uptake in SH-SY5Y.

Table S1: Cell uptake of NLs (µg) by hCMEC/D3 and SH-SY5Y in presence of transport inhibitors

	hCMEC/D3 cell line			SH-SY5Y cell line		
	NLb0	NLb1	NLb2	NLb0	NLb1	NLb2
			5 µg/mL			
4 °C	0.17±0.01	0.19±0.03	0.12±0.03	0.20±0.02	0.22±0.01	0.18±0.01
Chlorpromazine	0.13±0.01	0.20±0.02	0.08±0.01	0.23±0.02	0.23±0.01	0.20±0.01
Indomethacin	0.01±0.01	0.14±0.02	0.07±0.03	0.21±0.01	0.20±0.01	0.20±0.01
			10 µg/mL			
4 °C	0.44±0.01	0.44±0.08	0.39±0.04	0.53±0.05	0.47±0.02	0.46±0.03
Chlorpromazine	0.30±0.06	0.49±0.03	0.43±0.03	0.66±0.03	0.50±0.07	0.52±0.01
Indomethacin	0.29±0.04	0.44±0.10	0.49±0.06	0.68±0.02	0.58±0.03	0.38±0.01
			100 µg/mL			
4 °C	3.09±0.04	2.59±0.04	2.59±0.07	2.83±0.01	2.36±0.03	2.38±0.02
Chlorpromazine	3.43±0.10	3.03±0.09	3.01±0.024	3.77±0.15	3.27±0.20	2.73±0.06
Indomethacin	3.04±0.41	2.84±0.04	3.07±0.299	3.64±0.25	3.30±0.05	3.15±0.10

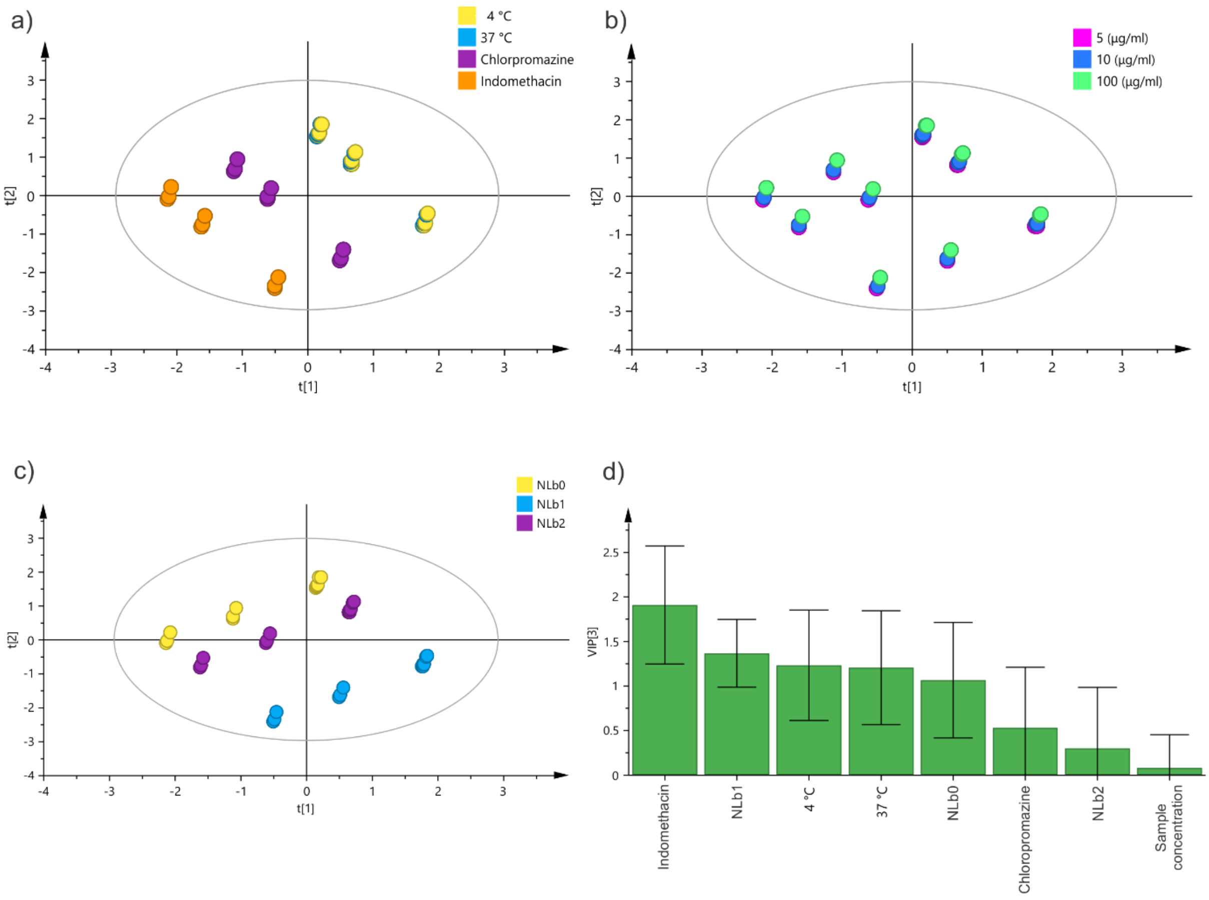

Figure S5: Score scatter plot colored by a) experimental conditions, b) sample concentration, c) formulation type, and d) VIP score for discriminative analysis of the factors affecting NL uptake in hCMEC/D3.

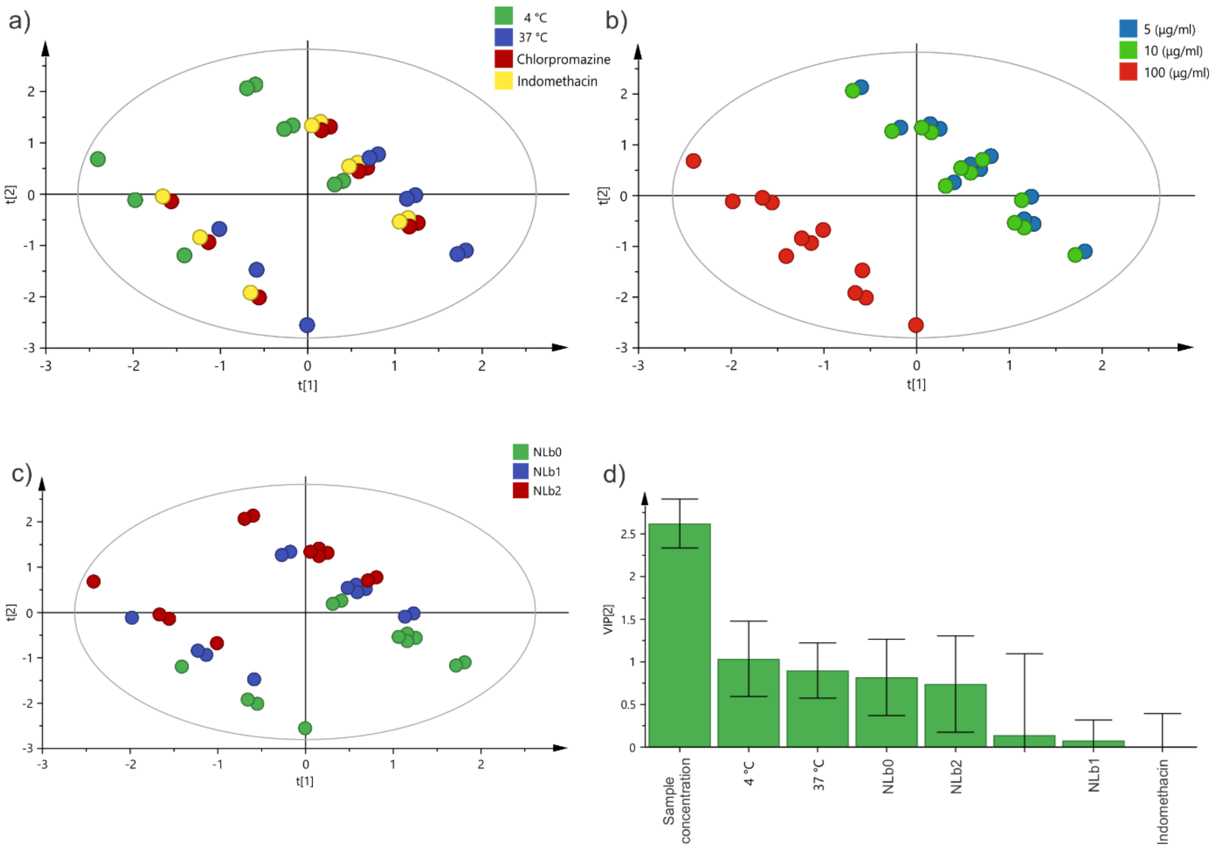

Figure S6: Score scatter plot colored by a) experimental conditions, b) sample concentration, c) formulation type, and d) VIP score for discriminative analysis of the factors affecting NL uptake in SH-SY5Y.
